# Phylogenetic assessment of alignments reveals neglected tree signal in gaps

**DOI:** 10.1186/gb-2010-11-4-r37

**Published:** 2010-04-06

**Authors:** Christophe Dessimoz, Manuel Gil

**Affiliations:** 1 Department of Computer Science, ETH Zurich, Universitaetstr. 6, 8092 Zürich, Switzerland; 2 Swiss Institute of Bioinformatics, Universitaetstr. 6, 8092 Zurich, Switzerland

## Abstract

**Background:**

The alignment of biological sequences is of chief importance to most evolutionary and comparative genomics studies, yet the two main approaches used to assess alignment accuracy have flaws: reference alignments are derived from the biased sample of proteins with known structure, and simulated data lack realism.

**Results:**

Here, we introduce tree-based tests of alignment accuracy, which not only use large and representative samples of real biological data, but also enable the evaluation of the effect of gap placement on phylogenetic inference. We show that (i) the current belief that consistency-based alignments outperform scoring matrix-based alignments is misguided; (ii) gaps carry substantial phylogenetic signal, but are poorly exploited by most alignment and tree building programs; (iii) even so, excluding gaps and variable regions is detrimental; (iv) disagreement among alignment programs says little about the accuracy of resulting trees.

**Conclusions:**

This study provides the broad community relying on sequence alignment with important practical recommendations, sets superior standards for assessing alignment accuracy, and paves the way for the development of phylogenetic inference methods of significantly higher resolution.

## Abstract 2

Tree-based tests of alignment methods enable the evaluation of the effect of gap placement on the inference of phylogenetic relationships.

## Background

The study of biological sequences almost inevitably begins with the process of alignment. The goal of this process is usually to match homologous characters, that is, characters that have a common ancestry [1]. In turn, these sets of homologs, the *columns *of the alignment, can be used for a variety of applications, such as identifying residues with analogous structural or functional role, or inferring the phylogenetic tree of the underlying sequences. The accuracy of multiple sequence alignment programs has been the object of numerous comparative studies [2-4], which evaluate alignments either by using trusted reference alignments obtained from structural data, or by using simulation. Unfortunately, both approaches have flaws. Trusted benchmark alignments such as Balibase, Prefab, Homstrad, or Sabmark [5-8] are all derived from protein structure information, exploiting the tendency of structure to evolve more slowly than sequence [9].

However, proteins with resolved structure remain a small and highly biased sample of all proteins [10,11]. In addition, homology inferred from structural information is inherently restricted to conserved regions, thereby providing little guidance for correct gap placement. The other approach to validating alignments is simulation [12-18]. Yet, results obtained from simulated data strongly depend on the choice of model used to generate the data, and most biological processes are difficult to model realistically. For instance, current insertion-deletion models are known to be insufficient [19]. Even if a good model can be formulated, it will never fully capture the complexity of real biological data. Consequently, the results observed on simulated data differ significantly from those measured on empirical data [1].

## Results and discussion

There is, therefore, a need for alternative evaluation procedures that do not rely on structural information while applicable to a large and representative sample of real biological data. In this work, we propose two such tests. We then show how they offer answers to three of the most important open questions regarding sequence alignment for phylogenetic inference: (i) Which alignment approach leads to the most accurate trees? (ii) Are gap regions informative for phylogenetic inference or should they be ignored? (iii) What is the impact of alignment uncertainty on tree inference?

### Phylogeny-based tests of alignment accuracy

**Figure 1:**
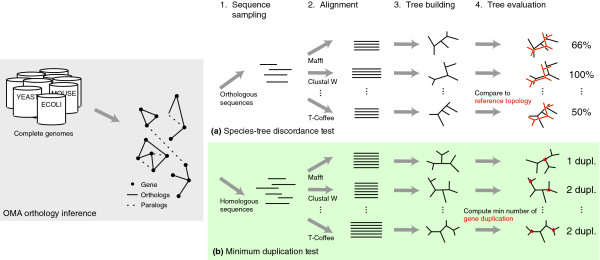
**Schematic of the phylogeny-based tests of alignment accuracy**. Both tests are based on large-scale genomic data: **(a) **The species-tree discordance test samples sets of orthologs inferred by OMA among species with a well-accepted phylogeny (Additional file 1, Figure S1). Each sample is aligned by the different packages. The resulting alignments are evaluated by reconstructing trees from them, and comparing with the reference topology. All else being equal, trees from better alignment packages show higher average congruence with the reference topology. **(b) **The minimum duplication test follows a similar idea, but differs from the first test in two ways. First, it samples sets of homologs rather than the more specific orthologs. Second, the evaluation is based on a parsimony argument rather than knowledge about the phylogeny of the species: all else being equal, alignments yielding trees with fewer duplication nodes on average are more accurate.

The principle of the phylogeny-based tests of alignment accuracy is simple: the more accurate the resulting trees, the more accurate the alignments (in terms of homology matching) are assumed to be. Therefore, we can use tree accuracy as surrogate for alignment accuracy. The first phylogeny-based test we propose ('species-tree discordance') compares alignments of orthologous genes from species whose phylogeny is resolved and undisputed (Figure 1). By Fitch's definition of orthology [20], trees inferred from orthologs are expected to have the same topology as the underlying species. Thus, holding all else constant, if a particular method produces alignments that result more frequently in trees congruent with the phylogeny of the species, it is likely to be more accurate. A similar idea was previously used in the context of model comparison [21], and verification of orthology [22]. The second test ('minimum duplication') takes homologous sequences as input and uses a parsimony argument rather than knowledge about the phylogeny of the species: holding all else constant, the gene tree with the least number of duplication nodes is the most likely (Figure 1, [23-25]). Hence, if a sequence alignment method results in tree topologies with consistently fewer duplications, it is likely to produce better alignments. Given a tree, a conservative estimate of the number of duplication events can be obtained using the concept of species overlap [26]. By accepting practically any gene family as input, the two tests can be performed on sequences relevant to a given biological study. Moreover, note that by design, the tests are robust to sources of errors that affect all alignment methods equally on average, such as stochastic errors in tree inference, lateral gene transfers, or the choice of evolutionary model. For instance, although the parsimony assumption may occasionally underestimate the true number of duplicated genes (for example, in gene families with many duplications/losses), as long as this underestimation does not favor a particular alignment method, the ranking of the methods is unaffected.

### Assessment of alignment methods

To address the question of alignment accuracy, we used the tests to evaluate 13 MSA software packages, which can be classified into roughly three alignment scoring strategies: *scoring matrix-based *Mafft FFT-NS-2, Muscle, Clustal W2, DiAlign/-T/-TX, Kalign [6,27-33]; *consistency-based *Mafft L-INS-i, T-Coffee, Mummals, ProbCons, ProbAlign [27,28,34-37]; and *tree-aware-gap-placing *Prank [38]. We tested the alignment software both on amino-acid and on nucleotide data, with the exception of Mummals and ProbCons, which only run on amino-acid data. For the species-tree discordance test, we sampled sets of 6 orthologs as inferred by OMA [39] among 57 eukaryotic, 11 fungal, and 418 bacterial genomes, under the constraint that the branching order of the species represented in each set be well-accepted (Additional file 1, Figure S1). For the minimum duplication test, we retrieved groups of up to 60 homologs from 18 metazoan and 18 fungal genomes. Trees were reconstructed by maximum likelihood (ML) from both amino-acid and nucleotide alignments. In addition, to compare the two types of alignments under the same evolutionary model, ML trees were also reconstructed from back-translated amino-acid alignments, using the actual codons from the corresponding nucleotide sequences. In total, the tests required computing over 100,000 alignments of up to 60 sequences, at a cost of over 20,000 CPU hours.

**Figure 2:**
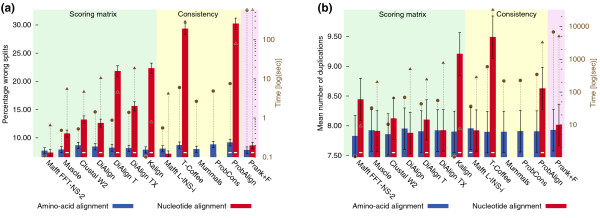
**Comparison of alignment methods**. Assessment of various alignment methods under default parameters using **(a) **the species-tree discordance and **(b) **the minimum duplication tests, on eukaryotic data. Consistency-based alignment methods do not improve over scoring matrix-based methods. The relative performance between alignment programs is more variable for nucleotide data than for amino-acid data. On amino-acid data, Mafft-FFT-NS-2, DiAlign TX and Prank were never outperformed; on nucleotide data, Mafft L-INS-i (right column) was never outperformed (see also Additional file 1, Figure S6). Average compute times (per alignment) are plotted as triangles (amino-acids) and circles (nucleotides). Error bars correspond to ± 1 s.d. Significant difference from best alignment program is denoted with a minus symbol at the basis of relevant bars (Wilcoxon double-sided test, *P *< 0.01).

In general, we observed fewer differences among programs aligning amino-acids than aligning nucleotides (Figure 2). Trees from nucleotide alignments fared significantly worse than those from back-translated amino-acid alignments in practically all cases. Since the only difference between the two types of trees resides in the alignment process, we conclude that current alignment packages align amino-acids more accurately than nucleotides (Additional file 1, Figure S7), as previously observed in simulation by [13]. In terms of alignment strategy, and contrary to current beliefs [3,4], consistency-based alignment methods as a class did not outperform their scoring matrix-based counterparts, yet they were up to 300 times slower (Figure 2, Additional file 1, Figure S6). Thus, the additional time spent by consistency-based programs did not necessarily translate into more accurate trees. In addition, the consistency-based methods surveyed here tended to perform unevenly across different datasets, which suggests that their underlying models and/or parameters are relatively sensitive to input data characteristics. The potential misguidance of current benchmarks is exemplified in the results obtained from the different versions of DiAlign: although both simulated and structure-based reference alignments indicated that DiAlign had significantly improved over the course of the three releases investigated here [32], the present tests do not support this conclusion. While significant differences among the versions can be observed in particular datasets, no DiAlign variant demonstrated superior performance. In terms of individual programs, only small differences could be observed with amino-acid sequences. It nonetheless appears that DiAlign TX and Prank were consistently among the best programs (Additional file 1, Figure S6). With nucleotide sequences, the differences were greater. Mafft L-INS-i was the only package consistently among the best on nucleotide data. At the other end of the spectrum, T-Coffee, KAlign and DiAlign T exhibited subpar nucleotide alignment performance. Overall, as we have seen that alignments are almost invariably more accurate on amino-acid data, the best nucleotide alignments are obtained by back-translating amino-acid alignments.

To limit the risk of systematic biases or unrecognized factors, these observations were confirmed by two kinds of controls. First, we considered the effect of the tree building method used in the test procedure. We ran the tests under a different model of evolution and using least squares distance trees instead of ML. The results were highly consistent (Additional file 1, Figures S8 and S9, relative accuracy of the two methods correlates with 0.90, *P *< 10^-10^, t-test). Second, we tested the dependence of the results on characteristics of the input data. We re-evaluated the tests on partitioned data and estimated the correlations between the relative accuracy of each partition with its full datasets. The data was segmented according to sequence length (Additional file 1, Figure S10, *r *= 0.62, *P *< 10^-10^), sequence divergence (Additional file 1, Figure S11, *r *= 0.67, *P *< 10^-10^) and number of sequences (Additional file 1, Figure S12, *r *= 0.89, *P *< 10^-10^). Furthermore, we contrasted the results of different pairs of lineages (Additional file 1, Figure S6, 0.68 <*r *≤ 0.94, all *P *< 10^-3^). In all cases, our conclusions above stand.

### Guide trees make or break progressive alignments

Since sequence insertion and deletion events are generally assumed to take place along a tree, most aligners rely on guide trees to construct and score alignments. Some of them - in our case Mafft, Muscle, Clustal W2, T-Coffee and Prank - allow specification of the guide tree by the user. To investigate their sensitivity to tree specification, we ran the species-tree discordance test on two extreme cases: we provided either a random guide tree, or the reference species tree as guide (Additional file 1, Figure S13). Unsurprisingly, the input trees hardly affected methods refining their guide trees iteratively (Muscle) or relying strongly on consistency (T-Coffee), a mostly tree-independent objective function. In contrast, strictly progressive methods (Mafft-FFT, Clustal W2, Prank) were highly sensitive to the provided guide tree. With such methods, guide tree specification is a double-edged sword: prior knowledge of the underlying sequence phylogeny, depending on its accuracy, can either improve the resulting alignments, or worsen them. Consequently, if the tree is known with high confidence, we recommend using it in conjunction with Prank or Mafft. If not, one might wonder which program infers the best guide trees, and whether feeding them to the other aligners could improve results overall. Our results suggest that on average, the best guide trees are inferred by Prank on amino-acid data, and Mafft on nucleotide data (Additional file 1, Figure S14). The difference is however not sufficiently large that the other alignment methods consistently profit from these improved guide trees (Additional file 1, Figure S15).

### Gaps carry substantial unexploited tree signal

**Figure 3:**
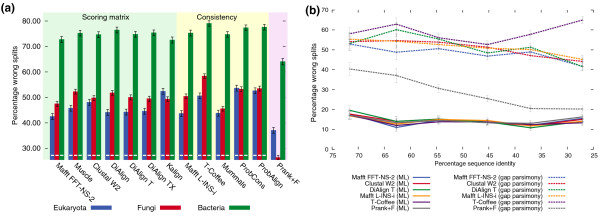
**Phylogenetic signal of gaps**. **(a) **Assessment of gap accuracy under default parameters using the species-tree discordance test with parsimony trees on presence/absence patterns of gap characters in aminoacid alignments. By taking into account gap information, this test demonstrates that the gap placement of Prank is significantly better than other alignment methods. This cannot be observed either using standard tree building methods (Figure 2), or using structure-based benchmarks. Error bars correspond to ± 1 s.d. Significant difference from Prank is denoted with a minus symbol at the basis of relevant bars (Wilcoxon double-sided test, *P *< 0.01). **(b) **Accuracy of maximum likelihood (ML) trees on amino-acid substitution patterns versus parsimony on binary gap presence/absence characters, on fungal data. The phylogenetic signal of gaps inferred by Prank increases with divergence. For distant sequences, the proportion of correctly inferred splits from gaps alone is close to that from amino-acids substitutions by ML. Thus, tree building methods could capture up to twice as much phylogenetic signal from the same data. Moreover, note that the crude approach used here to infer the gap trees likely understates the potential of gap patterns.

A notable advantage of our evaluation approach lies in its capacity to assess the accuracy and phylogenetic information content of gap regions. Given that structural alignments are inherently limited to regions of conserved structure, previous assessment of gap region accuracy were typically performed on simulated data only (for example, [40]). Using simulation, Löytynoja and Goldman, the authors of Prank, have recently argued that other alignment programs infer less phylogenetically plausible alignments [41]. However, though competitive, Prank did not show a clear advantage over the other alignment strategies in the tests described above, especially considering its much higher computational cost (Figure 2). As it turns out, this is mainly a consequence of gap treatment in current ML tree building methods: by modeling each gap position as unknown character, they ignore much of the phylogenetic signal from gaps. To assess the phylogenetic signal of gaps, we repeated our tests using a tree inference method that *only *uses gap signals: maximum parsimony on binary gap/no-gap characters. On amino-acid data, the results using gap parsimony trees clearly show that Prank outperforms the other programs regarding gap placement on real biological sequences, at times quite dramatically (Figure 3a). On nucleotide data, Prank was occasionally surpassed by one of the DiAlign variants, but showed solid performance overall (Additional file 1, Figure S16). More importantly, although parsimony trees obtained from gaps are on average much less accurate than ML trees from substitutions, with Prank, the difference between the two considerably diminishes, especially at high levels of sequence divergence (Figure 3b). In one extreme case (fungal nucleotide data, species-tree discordance test), the gap parsimony trees from alignments by Prank largely surpassed the ML trees from alignments by several other methods (Additional file 1, Figures S6 and S16). The broader implication of these results is that gaps carry significant phylogenetic signal, information that is currently ignored by most alignment and tree reconstruction programs (and certainly not completely exploited in the simplistic parsimony approach employed here). We stress that this unexpected result could only be observed by combining the recent improvements in alignment afforded by Prank, our alignment evaluation methods, and a tree inference procedure that exploits gap patterns.

### Excluding gaps and variable regions harms

**Figure 4:**
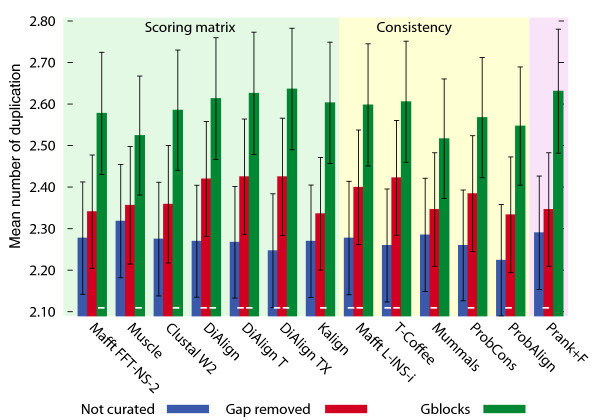
**Effect of excluding gaps and variable regions**. The plot shows the effect of filtering on the minimum duplication test with back-translated, fungal amino-acid alignments. Removing gapped sites tends to worsen the accuracy of the induced maximum likelihood trees. Removing variable regions in addition to gapped sites (Gblocks, default settings) drastically reduces the accuracy of reconstructed trees. Error bars correspond to ± 1 s.d. Significant difference between results from original and curated alignments is denoted with a minus symbol at the basis of relevant bars (Wilcoxon double-sided test, *P *< 0.01).

It has been argued that even if gap regions carry potential phylogenetic signal, inclusion of these regions, which are usually more difficult to align than conserved ones, results in an overall decrease in the signal-to-noise ratio of alignments [42]. And indeed, the common recommendation of excluding 'gaps and ambiguous sites' in phylogenetic analyses tends to support this view as well. Even so, in some cases, studies on particular gene families [43,44], or using simulation [18,45], have supported the opposite view. We investigated this issue by comparing trees reconstructed from full alignments versus from alignments without gap columns (that is, without columns containing gaps), and full alignments versus alignments curated by Gblocks [42]. By default, Gblocks identifies and removes both gap columns and variable regions. For amino-acid alignments, excluding gap columns never improved tree accuracy, and often worsened it (Figure 4, Additional file 1, Figures S18 and S19). Removing variable regions in addition to gaps, as performed by Gblocks, had a strong negative impact on the accuracy of trees. For nucleotide alignments, the effects were not nearly as detrimental; in some cases, the filtering helped (Additional file 1, Figure S19). But remember that alignment programs often have difficulty with nucleotide sequences; almost invariably, the best trees were obtained from unfiltered, amino-acid sequence alignments. Most striking about these findings is that, as pointed out above, the standard tree building methods used here do not exploit gap patterns; it appears that character substitution patterns inside gap and variable regions carry enough phylogenetic signal to warrant inclusion of those segments under current methods.

### Alignment variability poorly predicts tree accuracy

We have seen that different alignment programs can give rise to trees of varying accuracy. But in the broader context of tree inference, sequence alignment is not the only source of tree uncertainty. By 'uncertainty', we mean the expected addition of systematic and random error, that is, the expected inaccuracy. For instance, the amount of input data (that is, sequence lengths), the divergence between sequences, the model of evolution, or the tree searching algorithm all affect the accuracy of reconstructed trees, and one's confidence therein. This raises the question of the relative contribution of alignment uncertainty to tree uncertainty. Wong *et al*. recently quantified the observation that different alignment programs often lead to different tree topologies [46]. They found a correlation (Spearman-rank correlation *r*_*s *_= 0.53) between alignment variability (average distance between alignments from different methods) and tree variability (average topological distance among trees estimated from different alignment methods). But constrained by a lack of measure of total tree error, their analysis only focused on the random component of tree uncertainty. We exploited the tree accuracy measure from the species-tree discordance test to estimate the correlation between alignment variability and tree accuracy. Interestingly, accounting for both random and systematic errors suggests a weaker connection between alignment and tree quality: the negative correlation between alignment variability and tree accuracy was low for amino-acid and back-translated data (Additional file 1, Figure S20, -*r*_*s *_< 0.16, *P *< 0.01, t-test). Thus, alignment variability says little about overall tree uncertainty for amino-acid alignments. To put the results into perspective, we also estimated the correlation between bootstrap tree support and tree accuracy. Surprisingly, even though bootstrap *assumes correct alignments*, it was a consistently better predictor of tree accuracy than alignment variability (Additional file 1, Figure S20, *r*_*s*, *Bootstrap*_> -*r*_*s*, *AlignmentVar*_, *P *< 0.006, see methods). For nucleotide alignments, shown above to be often worse than amino-acid alignments, we found a higher correlation between alignment variability and tree accuracy than for the amino-acid counterparts. Still, alignment variability was never a better predictor of tree accuracy than tree support (Additional file 1, Figure S20). Since tree support is usually computed anyway, this casts doubt on the usefulness of trying more than one alignment method for the purpose of phylogenetic inference [47]. Rather, we recommend that practitioners stick with an accurate alignment method, as identified by tests such as the ones presented here.

## Conclusions

In summary, the use of trees rather than protein structure to assess alignments is advantageous in that it more closely fits a common application of alignments, it is not restricted to the relatively small and biased sample of proteins with known structure, and it also allows the evaluation of gap regions. Indeed, our results show that consistency-based alignment methods, which score best in structural benchmarks, do not yield significantly better trees than their scoring matrix-based counterparts. Our tests also demonstrate that gaps often carry a strong phylogenetic signal, which at present is not well exploited, either by most alignment methods, or by standard tree building methods; but even with such methods, excluding gaps and variable regions worsen the resulting trees. Finally, the low correlation we observed between alignment variability and tree accuracy suggests that there is little to gain from the common practice of trying more than one alignment program on a given dataset. This latter result, as well as the analysis on the impact of guide tree specification, rely exclusively on the species-tree discordance test, because they require knowledge of a reference topology. As such, the conclusions are based on six-taxa trees only. How well they generalize to larger trees is yet to be investigated. Besides, further interesting questions remain: how do alignment methods perform on data not represented in this study, such as promoter regions or other non-coding sequences? How can we best extend our current models of sequence evolution to take into account the phylogenetic signal of gap patterns? How do the methods investigated here compare with the statistical approach of joint alignment and tree inference? The methodology introduced here gives us the means to investigate these issues. Beyond alignments, the ability to measure tree accuracy under realistic conditions allows assessment of further important aspects of phylogeny inference, such as evolutionary models, tree building algorithms, or tree confidence measures.

## Materials and methods

### Sets of orthologous protein sequences

The Species Tree Discordance Test was performed on three sets of species: eukaryotes, fungi, and bacteria (detailed list in *Supplementary Information *Sect. 1.1). For all three sources of data, we retrieved sets of orthologs as inferred by OMA (Release of September 2008) [48]. Although cases of misclassification cannot be excluded, it has been shown in a previous study that the false-positive rate of OMA's predictions is low compared with other similar projects [22]. More importantly, though the presence of non-orthologs reduces the power of our test, it does not bias the results toward a particular alignment program. Sequences were sampled according to reference trees with a comb topology (Additional file 1, Figure S1). This topology ensures that all sequences in a sample are orthologous to each other [22]. In each trial, a starting sequence from a random species in the innermost leaf was randomly chosen. Then, for each remaining leaf, a random orthologous sequence was sampled.

### Sets of homologous protein sequences

We performed the Minimum Duplication test on two sets of organisms: metazoa and fungi (detailed list in *Supplementary Information *Sect. 1.2). Sets of homologs were constructed by taking the transitive closure of pairs of sequences with high alignment scores (E-value below 10^-10^). The sets were restricted to a maximal size of 60 sequences by removing sequences randomly from sets of excessive cardinality.

### Definition: absolute minimum number of duplications

For any set of homologous genes, consider partitions of the sequences according to their genome of origin: each resulting partition consists of same-species paralogs. Let *m *be the maximum cardinality of these partitions. For *m *paralogs to be observed in the same genome, at least *m-*1 duplications had to take place. We denote *m-*1 as *absolute minimum number of duplications *for the set of homologs.

### Species-tree discordance test

The species-tree discordance test evaluates a sequence alignment program in terms of the average accuracy of the trees reconstructed from its alignments. The test requires a large number of sequence sets whose phylogeny is known. Given that orthologous genes (by definition) follow the species tree, we sampled orthologs provided by OMA [48] from species with known and undisputed branching order (Additional file 1, Figure S1). Agreement between obtained and reference topologies was quantified by the proportion of wrong splits [49].

### Minimum duplication test

In a gene tree, the split of two same-species paralogs is necessarily a duplication event. By a parsimonious argument, the tree with the least duplication splits represents the most likely evolutionary history. The minimum duplication test evaluates a sequence alignment program in terms of the average minimum number of gene duplication events implied in the trees reconstructed from its alignments of homologous sequences. Given a rooted tree, a lower bound on the number of duplications can be obtained by counting nodes that have subtrees with overlapping sets of species [26]. Since the placement of the root of the tree is usually unknown, we considered all possible rootings and retained the minimum number of duplications. This measure was normalized by subtracting the *absolute minimum number of duplications *from it (see above). An example computation can be found in Additional file 1, Figure S2.

### Tree reconstruction

Gene trees were reconstructed by maximum likelihood using PhyML v. 2.4.4 [50] from the sequences aligned with the different programs under JTT+I+Γ for amino-acids and HKY+I+Γ for nucleotides. To investigate the accuracy of gap placement, the two tests were also performed using Wagner parsimony on the presence/absence patterns of gaps (for a given alignment, each column containing at least one gap was considered a character and the presence/absence of a gap its state). To avoid over-counting, neighboring columns with identical gap-patterns were combined into single characters.

### Alternative tree building methods

As control, we recomputed the trees using a least-square distance approach instead of maximum likelihood: we reconstructed variance weighted least-squares distance trees using the *MinSquareTree *function in Darwin [51]. The pairwise input-distances were computed by maximum likelihood using the GCB matrices [52] for amino-acid data. For nucleotide data we used an unpublished, empirical nucleotide substitution matrix estimated from mammalian orthologs in OMA [48]. Likewise, as an alternative (and control), we recomputed the Gap Parsimony Trees *without *combining repeated columns. Furthermore, for a subset of the tests we repeated the computation of the ML trees using the software RAxML v. 7.0.4 [53].

### Filtering of gaps and variable regions

We define a gap column as a column of the multiple sequence alignment in which at least one sequence has a gap character. To filter both gaps and variable regions, we used Gblocks version 0.91b [42] with default settings. In addition and as control, we also relaxed the settings according to Talavera *et al*. [42]. At times, any of the three filtering variants (no gap, Gblocks default, Gblocks relaxed) could yield alignments with no column left, that is, of null length. Such samples were excluded.

### Measures to relate alignment uncertainty to tree inference

The measures used in the section *Alignment Variability Poorly Predicts Tree Accuracy *and Additional file 1, Figure S18 are defined as follows: *Tree accuracy *was measured by one minus the normalized Robinson-Foulds distance [49] between the inferred and the accepted topology. *Tree support *was measured by the proportion of bootstrap replicates agreeing with the inferred topology. *Tree variability *was measured by the average Robinson Foulds distance among trees estimated from different alignment methods. *Alignment variability *was measured by the average distance between alignments [54] from different alignment methods. This measure has been shown [46] to strongly correlate (Spearman's rank correlation *r*_*s *_= 0.92, *P *< 0.0001) with Bayesian-inferred alignment variability.

### Comparing two correlation coefficients

We have stated in the main text (see also Additional file 1, Figure S18) that tree support (*BS*) is a better predictor for tree accuracy (*TA*) than alignment variability (*AV*). This can be assessed by the following test: As a null hypothesis, equal predictive power of the two measures is assumed, that is *r*_*s*_(*BS, TA*) = -*r*_*s*_(*AV, TA*). The observation (Additional file 1, Figure S18) that for all datasets *r*_*s*_(*BS, TA*) > -*r*_*s*_(*AV, TA*) is formulated as an alternative hypothesis. We assume that the pair samples are normal bivariate distributed.

is approximately standard normal distributed, where *z*(·) denotes the Fisher Z-transform.

## Authors' contributions

CD and MG contributed equally to this work.

## Supplementary Material

Additional file 1 **Supplementary information**. A 34-page PDF file with (1) description of software and sequence data and software, in particular supplementary figures S1 to S5; (2) an example for the computation of the evaluation criterion in the Minimum Duplication Test; (3) additional support and controls for the results presented in the main text, mainly consisting of supplementary figures S6 to S24; (4) description of the raw results, which can be downloaded in their entirety. Click here for file
