# Supplementary material for: Phylogenetic assessment of alignments reveals neglected tree signal in gaps
Source: Genome Biol. 2010 Apr 6;11(4):R37. doi: 10.1186/gb-2010-11-4-r37 (PMC2884540; doi:10.1186/gb-2010-11-4-r37)
Supplement: Additional file 1 — Supplementary information. A 34-page PDF file with (1) description of software and sequence data and software, in particular supplementary figures S1 to S5; (2) an example for the computation of the evaluation criterion in the Minimum Duplication Test; (3) additional support and controls for the results presented in the main text, mainly consisting of supplementary figures S6 to S24; (4) description of the raw results, which can be downloaded in their entirety. [file gb-2010-11-4-r37-S1.PDF]

Supplementary Information for "Phylogenetic Assessment of Alignments Reveals Neglected Tree Signal in Gaps"

Christophe Dessimoz* and Manuel Gil

ETH Zurich, Department of Computer Science, CH-8092 Zürich
and Swiss Institute of Bioinformatics

*To whom correspondence should be addressed; E-mail: cdessimoz@inf.ethz.ch

This document is structured as follows. Part 1 provides details on the input sequence data used and contains an example for the computation of the evaluation criterion in the Minimum Duplication Test. Part 2 provides additional support for the results presented in the main text. This part mainly consists of additional plots and tests, which are ordered according to the main text. Finally, Part 3 provides a description of the raw results, which are made available in their entirety.

1 Material

1.1 Accepted Topologies and Lists of Species for Species-tree Discordance Test

The Species-tree Discordance Test was performed on three sets of species: eukaryotes, fungi, and bacteria. SFig. 1 depicts the topology used for the reference trees. The numbers at the leaves map to the lists below, of which one orthologous sequence was selected for each sample. The five letter codes used for species identification are available at

http://www.cbrg.ethz.ch/research/msa

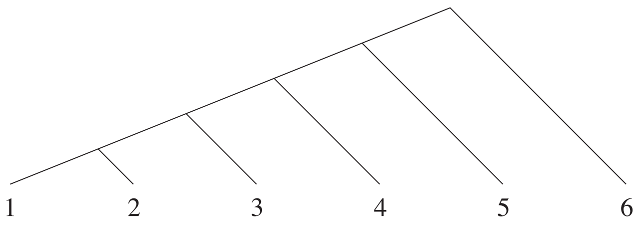

SFig. 1: Reference topology for the species-tree discordance test. The numbers at the leaves map to lists of species given in the text.

Eukaryota:

1. *Homo sapiens:* HUMAN
2. *Other Primates:* MACMU, MICMU, OTOGA, PANTR, PONPA
3. *Other Mammalia:* BOVIN, CANFA, CAVPO, DASNO, ECHTE, ERIEU, FELCA, HORSE, LOXAF, MONDO, MOUSE, MYOLU, OCHPR, ORNAN, RABIT, RATNO, SORAR, SPETR, TUPGB
4. *Other Vertebrata:* CHICK, DANRE, FUGRU, GASAC, ORYLA, TETNG, XENTR
5. *Protostomia:* AEDAE, ANOGA, APIME, DAPPU, DROME, DROPS, HELRO, LOTGI
6. *Fungi:* ASHGO, ASPFU, BOTFB, CANAL, CANGA, CRYNE, DEBHA, ENCCU, KLULA, LODEL, MAGGR, PHANO, PICST, SCHPO, USTMA, YARLI, YEAST

Fungi:

1. CANGA, YEAST
2. ASHGO, KLULA
3. CANAL, DEBHA
4. YARLI
5. ASPFU, SCHPO
6. CRYNE, ENCCU

Bacteria:

1. *Gammaproteobacteria:* ACIAD, ACIBT, ACTP2, ACTSZ, AERHH, AERS4, ALCBS, ALHEH, BAUCH, BLOFL, BLOPB, BUCAI, BUCAP, BUCBP, BUCCC, CARRP, CHRSD, COLP3, COXBU, DICNV, ECO24, ECO57, ECODH, ECOK1, ECOL5, ECOL6, ECOLI, ECOUT, ENT38, ERWCT, FRAT1, FRATF, FRATH, FRATN, FRATO, FRATT, FRATW, HAEDU, HAEI8, HAEIE, HAEIG, HAEIN, HAES1, HAHCH, HALHL, IDILO, KLEP7, LEGPA, LEGPC, LEGPH, LEGPL, MANSM, MARAV, MARMS, METCA, NITOC, PASMU, PHOLL, PHOPR, PSE14, PSEA6,

PSEA7, PSEAB, PSEAE, PSEE4, PSEF5, PSEHT, PSEMY, PSEP1, PSEPF, PSEPG, PSEPK, PSEPW, PSESM, PSEU2, PSEU5, PSYAR, PSYCK, PSYIN, PSYWF, RUTMC, SACD2, SALAR, SALCH, SALPA, SALTI, SALTY, SERP5, SHEAM, SHEB5, SHEB8, SHEB9, SHEDO, SHEFN, SHEHH, SHELP, SHEON, SHEPA, SHEPC, SHESA, SHESH, SHESM, SHESR, SHESW, SHIBS, SHIDS, SHIF8, SHIFL, SHISS, SODGM, STRMK, THICR, VESOH, VIBCH, VIBF1, VIBPA, VIBVU, VIBVY, WIGBR, XANAC, XANC5, XANC8, XANCB, XANCP, XANOM, XANOR, XYLFA, XYLFT, YERE8, YERP3, YERPA, YERPE, YERPN, YERPP, YERPS

2. *Betaproteobacteria:* ACIAC, ACISJ, AZOSB, AZOSE, BORA1, BORBR, BORPA, BORPD, BORPE, BURCA, BURCH, BURCM, BURM7, BURMA, BURP0, BURP1, BURP6, BURPS, BURS3, BURTA, BURVG, BURXL, CHRVO, DECAR, HERAR, JANMA, METFK, METPP, NEIG1, NEIM0, NEIMA, NEIMB, NEIMF, NITEC, NITEU, NITMU, POLNA, POLSJ, POLSQ, RALEH, RALEJ, RALME, RALSO, RHOFD, THIDA, VEREI

3. *Alphaproteobacteria:* AGRT5, ANAMM, ANAPZ, AZOC5, BARBK, BARHE, BARQU, BRAJA, BRASB, BRASO, BRUA2, BRUAB, BRUME, BRUO2, BRUSI, BRUSU, CAUCR, EHRCJ, EHRCR, EHRRG, EHRRW, ERYLH, GLUDA, GLUOX, GRABC, HYPNA, JANSC, MAGMM, MARMM, MESSB, NEOSM, NITHX, NITWN, NOVAD, OCHA4, ORITB, PARDP, PARL1, PELUB, RHIEC, RHIL3, RHILO, RHIME, RHOP2, RHOP5, RHOPA, RHOPB, RHOPS, RHORT, RHOS1, RHOS4, RHOS5, RICAH, RICB8, RICBR, RICCK, RICCN, RICFE, RICM5, RICPR, RICRO, RICRS, RICTY, ROSDO, SILPO, SILST, SINMW, SPHAL, SPHWW, WOLPM, WOLTR, ZYMMO

4. *Deltaproteobacteria:* ANADE, ANADF, BDEBA, DESDG, DESPS, DESVH, DESVV, GEOMG, GEOSL, GEOUR, LAWIP, MYXXD, PELCD, PELPD, SORC5, SYNAS, SYNFM
   and Epsilonproteobacteria: ARCB4, CAMC1, CAMC5, CAMFF, CAMJ8, CAMJD, CAMJE, CAMJJ, CAMJR, HELAH, HELHP, HELPH, HELPJ, HELPY, NITSB, SULDN, SULNB, WOLSU

5. *Spirochaetes:* BORAP, BORBU, BORGA, LEPBJ, LEPBL, LEPIC, LEPIN, TREDE, TREPA

6. *Firmicutes:* ACHLI, ALKMQ, ALKOO, AYWBP, BACA2, BACAH, BACAN, BACC1, BACCN, BACCR, BACCZ, BACHD, BACHK, BACLD, BACSK, BACSU, BACWK, CARHZ, CLOAB, CLOB1, CLOB8, CLOBH, CLOBK, CLOD6, CLOK5, CLONN, CLOP1, CLOPE, CLOPH, CLOPS, CLOTE, CLOTH, DESHY, ENTFA, EXIS2, GEOKA, GEOTN, LACAC, LACBA, LACC3, LACDA, LACDB, LACGA, LACH4, LACJO, LACLA, LACLM, LACLS, LACPL, LACRF, LACS1, LACSS, LEUMM, LISIN, LISMF, LISMO, LISW6, MESFL, MOOTA, MYCCT, MYCGA, MYCGE, MYCH2, MYCH7, MYCHJ, MYCMO, MYCMS, MYCPE, MYCPN, MYCPU, MYCS5, NATTJ, OCEIH, OENOB, ONYPE, PEDPA, STAA1, STAA2, STAA3, STAA8, STAA9, STAAB, STAAC, STAAE, STAAM, STAAN, STAAR, STAAS, STAAT, STAAW, STAEQ, STAES, STAHJ, STAS1, STRA1, STRA3, STRA5, STRGC, STRMU, STRP1, STRP2, STRP3, STRP6, STRP8, STRPB, STRPC, STRPD, STRPF, STRPG, STRPM, STRPN, STRR6, STRS2, STRSV, STRSY, STRT1, STRT2, STRTD, SYNWW, THETN, UREPA

1.2 Lists of Species for Minimum Duplication Test

The test was performed on two sets of organisms: metazoa (BOVIN, CAEBR, CAEEL, CAERE, CHICK, CIOSA, DAPPU, ECHTE, ERIEU, HUMAN, LOTGI, LOXAF, MONDO, MOUSE, NEMVE, PANTR, RABIT, XENTR) and fungi (ASHGO, ASPFU, BOTCI, BOTFB, CANAL, CANGA, CRYNE, DEBHA, ENCCU, KLULA, LODEL, MAGGR, PHANO, PICST, SCHPO, USTMA, YARLI, YEAST).

1.3 Example of Tree Evaluation

SFig. 2 shows how the minimum duplication test can score trees.

1.4 Alignment packages under consideration

The following packages were tested with default parameters, unless otherwise indicated:

- *Mafft 6.611 beta*. We tested both the L-INS-i option for accurate alignments and the FFT-NS-2 option for fast alignments, as described in (27,28).
- *Muscle 3.6* (6)
- *Clustal W 2.0.09* (29)
- *DiAlign 2.2.1* (30)
- *DiAlign-T 0.2.2* (31)
- *DiAlign-TX 1.0.1* (32)
- *Kalign 2.03* (33)
- *T-Coffee 5.72* (34)
- *ProbAlign 1.1 beta* (37)

- *Prank 02.12.08* (38) It has been shown by simulation to give more accurate results than the default settings.
- *Mummals 1.01* (35)
- *ProbCons 1.12* (36)

Mummals and ProbCons are not designed to align nucleotide sequences and therefore could only run on amino-acid data.

1.5 Characterization of Input Data

We have characterized the input data in terms of number of sequences, sequence-length and percentage sequence identity (minimum duplication test: SFig. 3, species-tree discordance test: SFig. 4). Additionally, SFig. 5 shows the distribution of the absolute minimum number of duplications in the species-tree discordance test.

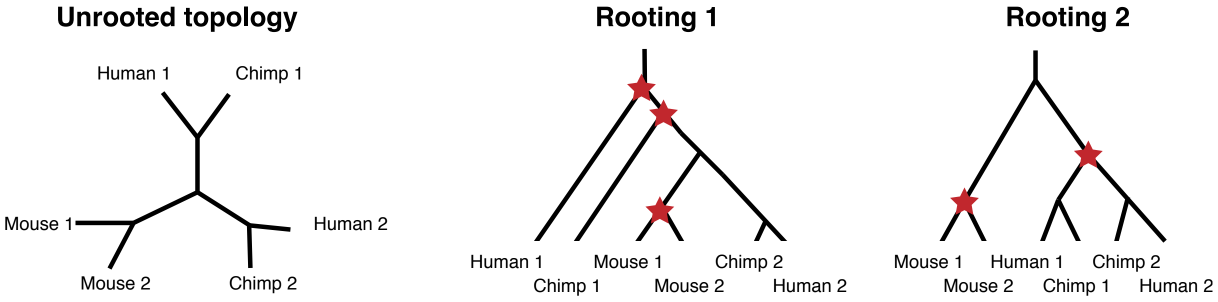

(a)

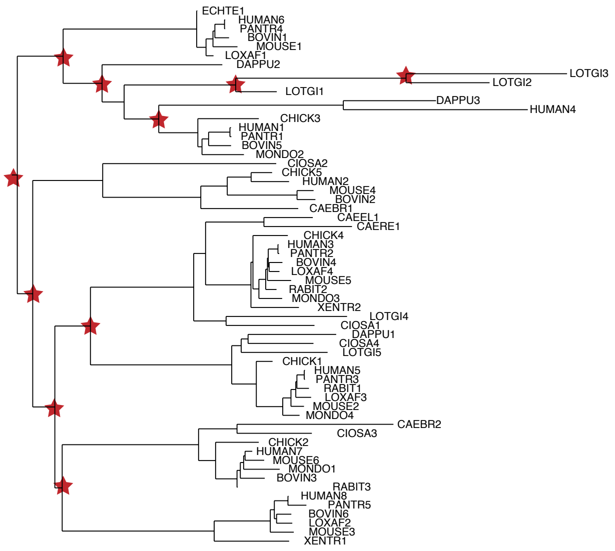

(b)

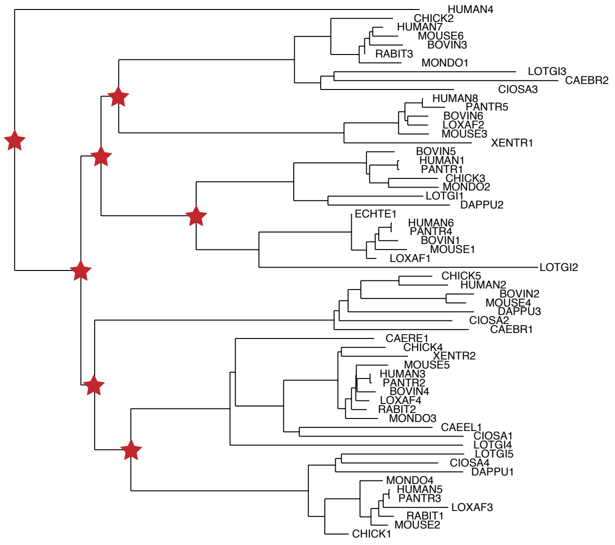

(c)

SFig. 2: Example of tree evaluation in minimum duplication test. (a) The set of homologs contains two sequences from human, chimp and mouse. The absolute minimum number of duplications n is 1. To score the obtained rooted topology (left), we consider all rootings, of which we depict two here: rooting 1 (middle) has 3 duplication splits (red stars), while rooting 2 (right) has 2 duplications. Knowing that there is no other rooting with fewer duplications than rooting 2, the final score is $2 - n = 1$. (b) This GDP-fucose transporter gene tree, inferred from sequences aligned by Mafft FFT-NS-2, requires 10 duplications. Since human – the species with most copies – has 8 of them, the minimum number of duplication is 7, and the tree score is $10 - 7 = 3$. (c) The same sequences aligned by Mafft-L-INS-i resulted in a tree with 7 duplications, for a final tree score of $7 - 7 = 0$. Based on this single example, the MD test would conclude that Mafft-L-INS-i is the more accurate aligner.

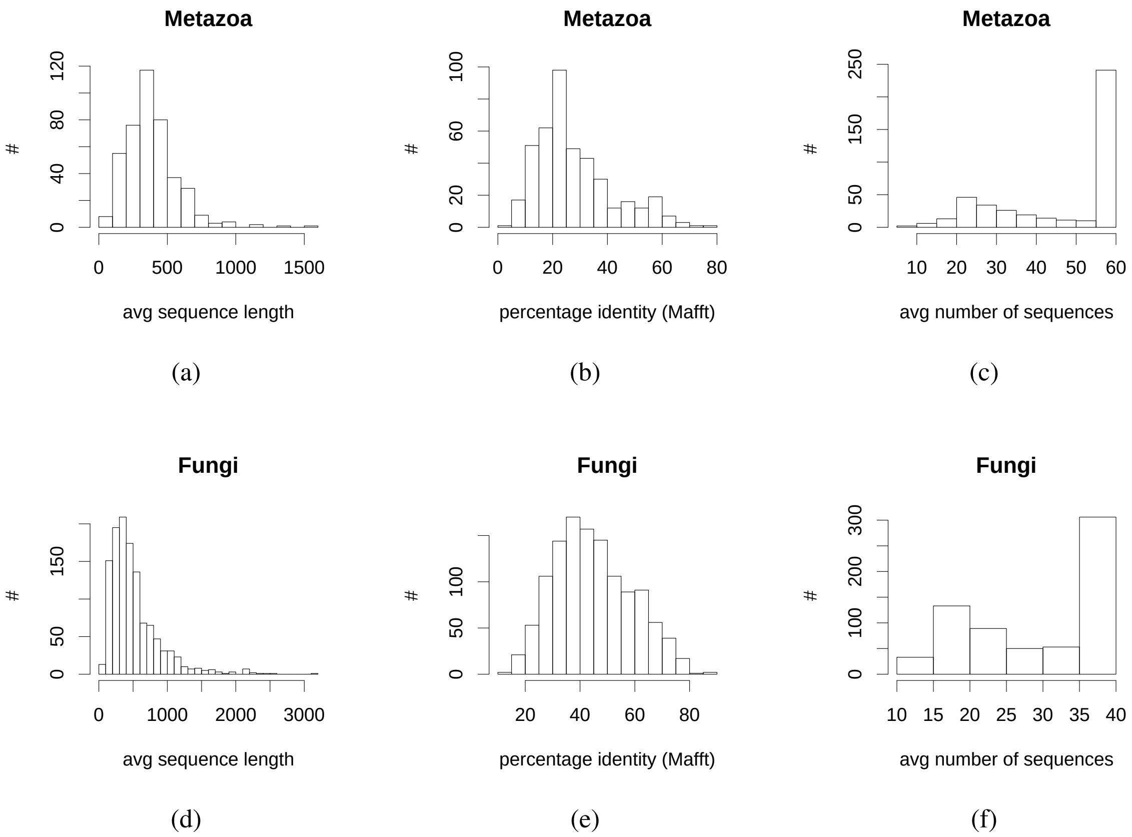

SFig. 3: Characterization of input data for minimum duplication test by amino-acid sequence length (left), divergence in percentage identity of the amino-acid sequences (middle) and number of sequences (right), for metazoa (top row) and fungi (bottom row). The percentage of sequence identity was computed using alignments by Mafft. Computing it from alignments by Prank resulted in very similar distributions.

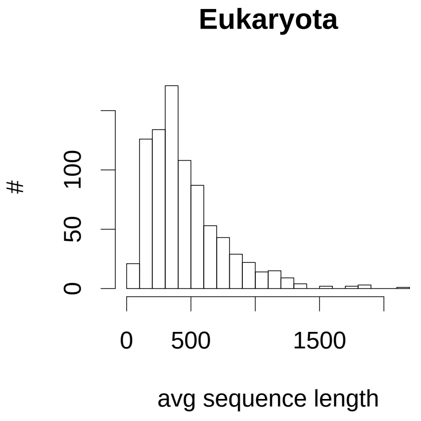

(a)

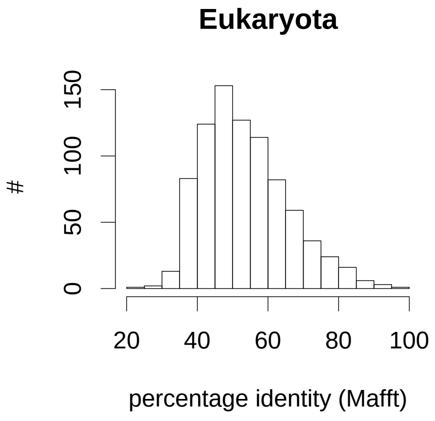

(b)

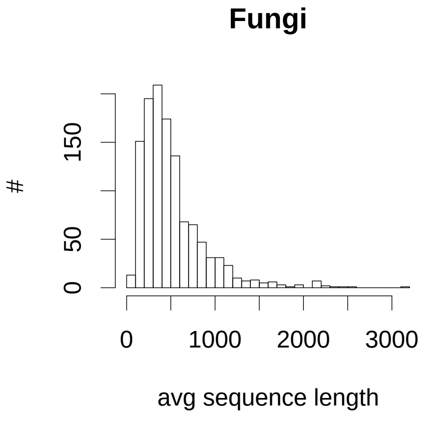

(c)

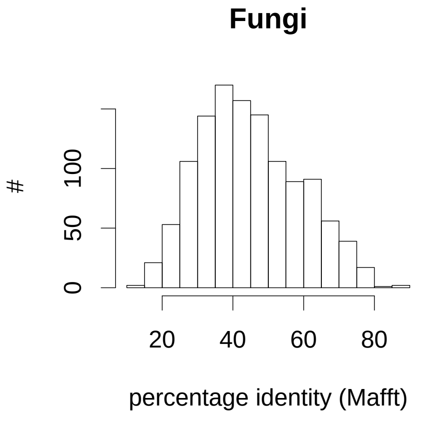

(d)

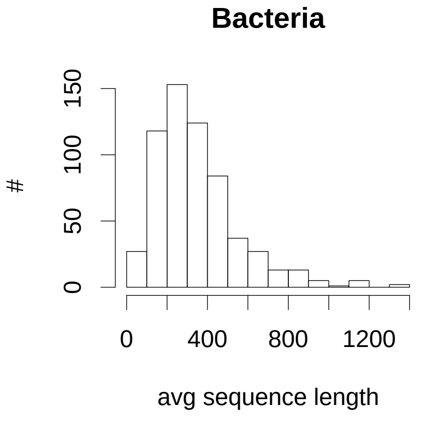

(e)

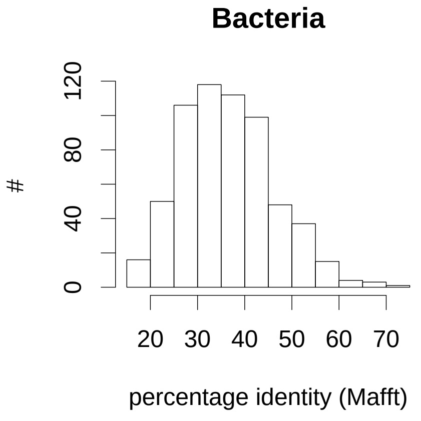

(f)

SFig. 4: Characterization of input data for species-tree discordance test by amino-acid sequence length (left), divergence in percentage identity of the amino-acid sequences (right) for eukaryotes (top row), fungi (middle row), and bacteria (bottom row). The number of sequences in all samples is 6. The percentage of sequence identity was computed using alignments by Mafft. Computing it from alignments by Prank resulted in very similar distributions.

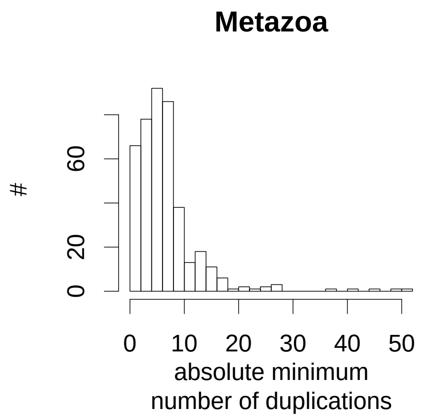

(a)

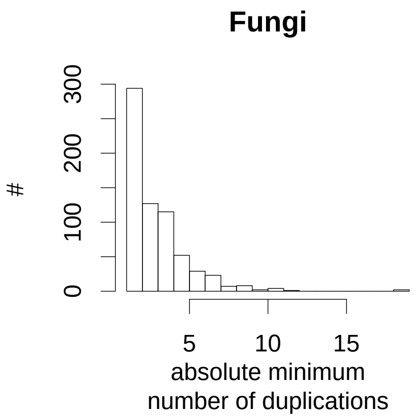

(b)

SFig. 5: Absolute minimum number of duplications for Metazoa (left) and Fungi (right) datasets.

2 Results: Additional Figures and Controls

The material in this section is ordered according to the main text. It consists of additional results and controls.

2.1 Which alignment approach or software package performs best?

- *Datasets with Other Species.* SFig. 6 shows that the test results using eukaryotic sequences (Fig. 2) hold with fungal and bacterial sequences as well.

- *Consistency in Ranking Between Trees from Amino-Acid, Back-translated, and Nucleotide Sequences.* SFig. 7 compares results from amino-acid, back-translated, and nucleotide sequences. It shows that alignment packages align amino-acid sequences more accurately than nucleotide sequences, and by including both amino-acid and back-translated results, it verifies that the results are not biased by the evolutionary model used in the tree inference.

- *Insensitivity to Tree-building Method.* The species-tree discordance test, originally performed with maximum likelihood tree construction, was repeated for least squares distance trees (see *Methods* for a description). The relative performance of the alignment packages was preserved under the two tree building methods.

- *Input Data Partition Analysis.* We partitioned the results reported in Fig. 2 in terms of the average sequence length (SFig. 10), the average divergence (SFig. 11) and the number of sequences (SFig. 12). The results from the partitioned data and from the full data were consistent.

In the main text, we report a number of correlations summarizing these controls. The correlation between two datasets was computed from pairs of average performance obtained under the same alignment programs (either % wrong splits or minimum number of duplication). Prior

to the correlation estimation, the accuracy measures from different datasets were normalized as follows:

$$\frac{x_i - \mathrm{avg}(\{x_j\})}{\mathrm{avg}(\{x_j\})},$$

where x_i denotes the accuracy of a particular alignment method i and $\mathrm{avg}(\{x_j\})$ the average accuracy of all methods considered in the dataset in question. All correlations were computed over both amino-acid and nucleotide results. For correlations between different pairs of lineages, we only report the range in the main text; the following table provides all values.

		correlation	p-value
Minimum Duplication Tree:	Metazoa vs. Fungi	0.9425	$< 10^{-10}$
Species-Tree Discordance:	Eukaryotes vs. Bacteria	0.7085	$1.0 \cdot 10^{-4}$
	Eukaryotes vs. Fungi	0.6860	$2.1 \cdot 10^{-6}$
	Fungi vs. Bacteria	0.8082	$1.7 \cdot 10^{-6}$

Fungal and Bacterial Datasets

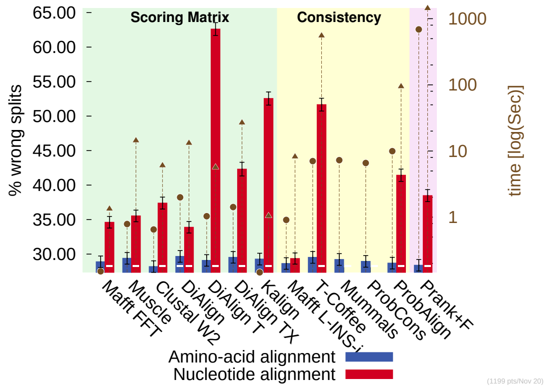

(a) Species-Tree Discordance, Fungi

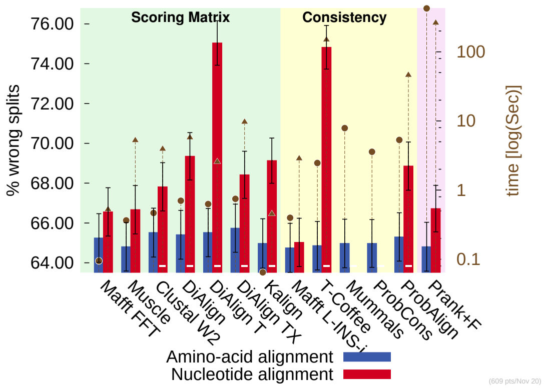

(b) Species-Tree Discordance, Bacteria

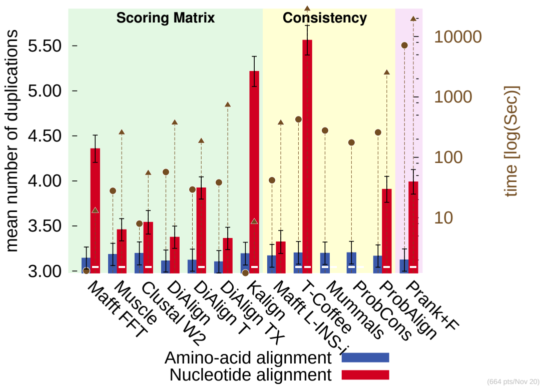

(c) Minimum Duplication, Fungi

SFig. 6: Species-tree discordance and minimum duplication test on additional datasets. Significant difference from fastest alignment program is denoted with a minus symbol at the basis of relevant bars (Wilcoxon double-sided test, $P<0.01$)

Comparison between amino-acid, back-translated, and nucleotide data

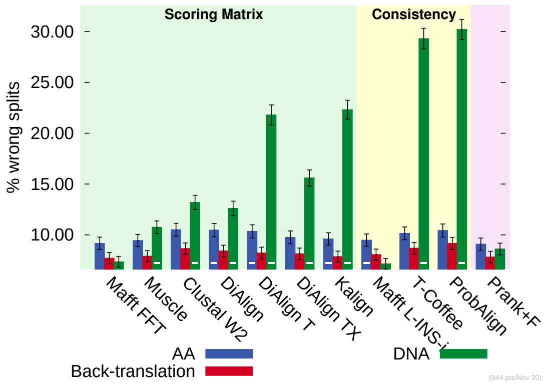

(a) Species-Tree Discordance, Eukaryotes

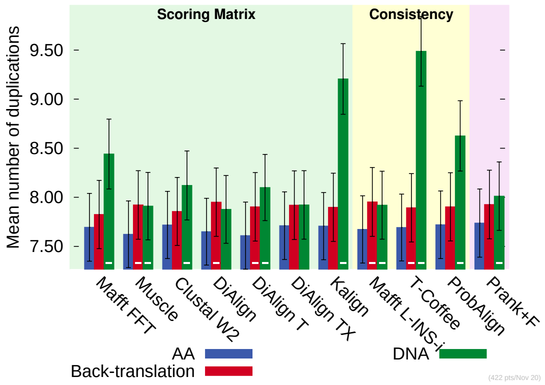

(b) Minimum Duplication Test, Metazoa

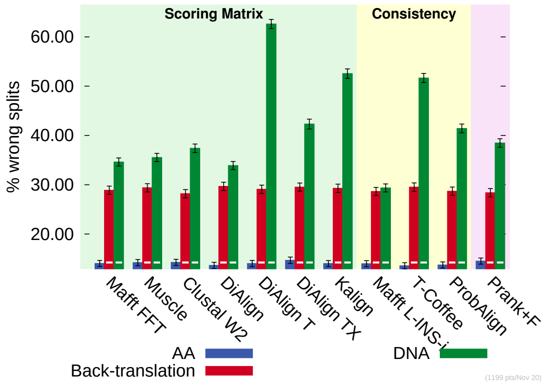

(c) Species-Tree Discordance, Fungi

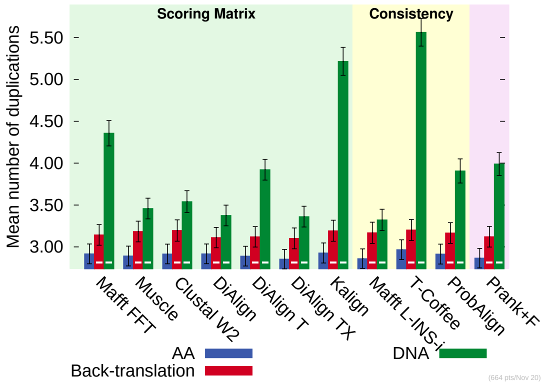

(d) Minimum Duplication Test, Fungi

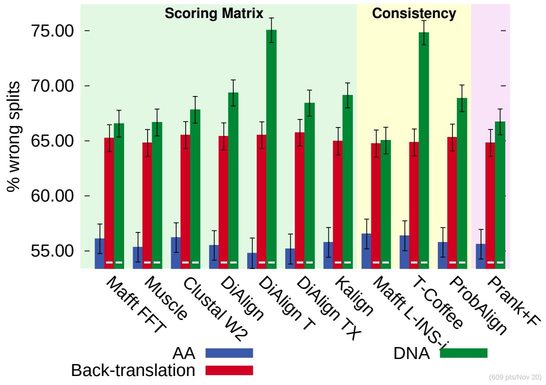

(e) Species-Tree Discordance, Bacteria

SFig. 7: Comparison between amino-acid, back-translated, and nucleotide data. First, the ranking of alignment methods is the same under both amino-acid and back-translated data (derived from common alignments); this further confirms that the test results are not affected by the tree reconstruction method. Second, the large difference between back-translated and nucleotide data stems exclusively from the alignment process, which is clearly worse on nucleotide data. Error bars correspond to $\pm$ 1 s.d. Significant difference from best data type is denoted with a minus symbol at the basis of relevant bars (Wilcoxon double-sided test, P<0.01)

Insensitivity to tree building method: species-tree discordance test

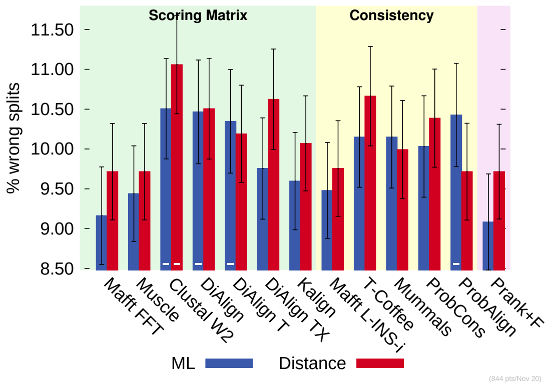

(a) Eukaryotes, Amino-acids

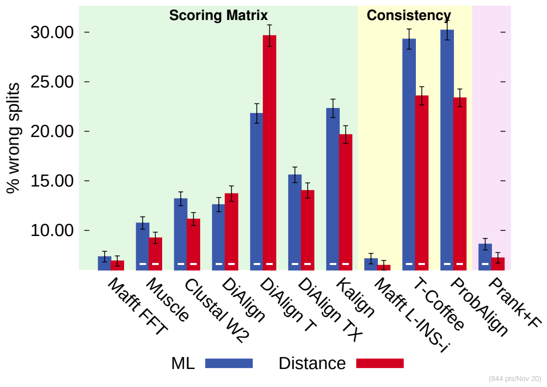

(b) Eukaryotes, Nucleotides

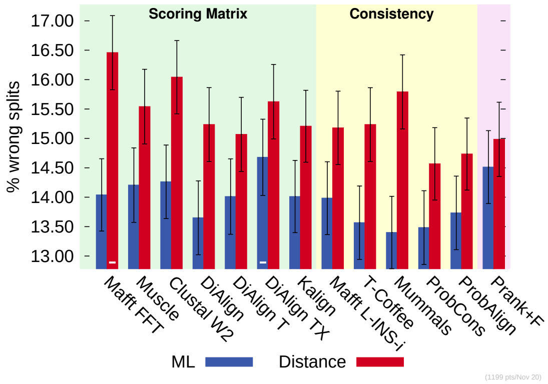

(c) Fungi, Amino-acids

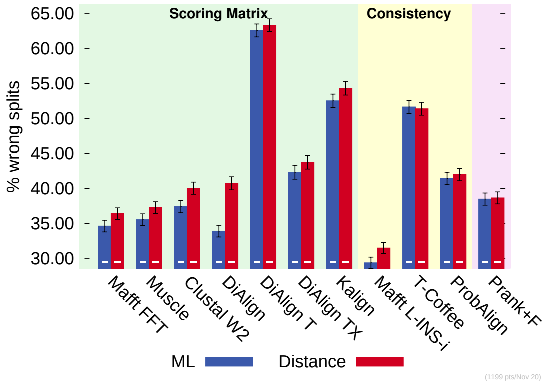

(d) Fungi, Nucleotides

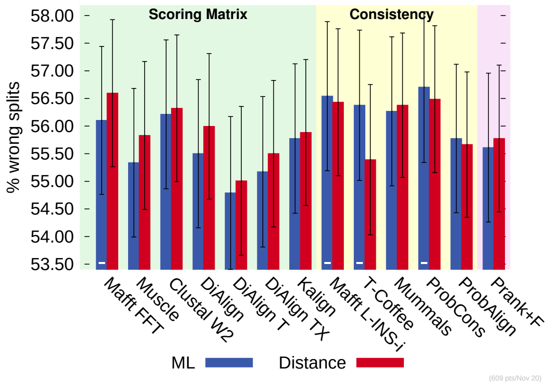

(e) Bacteria, Amino-acids

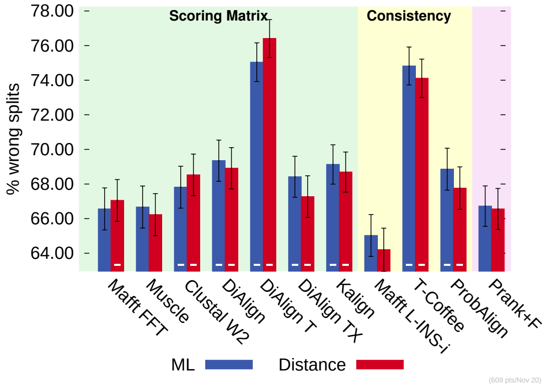

(f) Bacteria, Nucleotides

SFig. 8: Species-tree discordance test. The ranking of the alignment methods remains largely unchanged under the ML and the LS tree building approach. Error bars correspond to ± 1 s.d. Significant difference from the best alignment program is denoted with a minus symbol at the basis of relevant bars(Wilcoxon double-sided test, P<0.01)

Insensitivity to tree building method: minimum duplication test

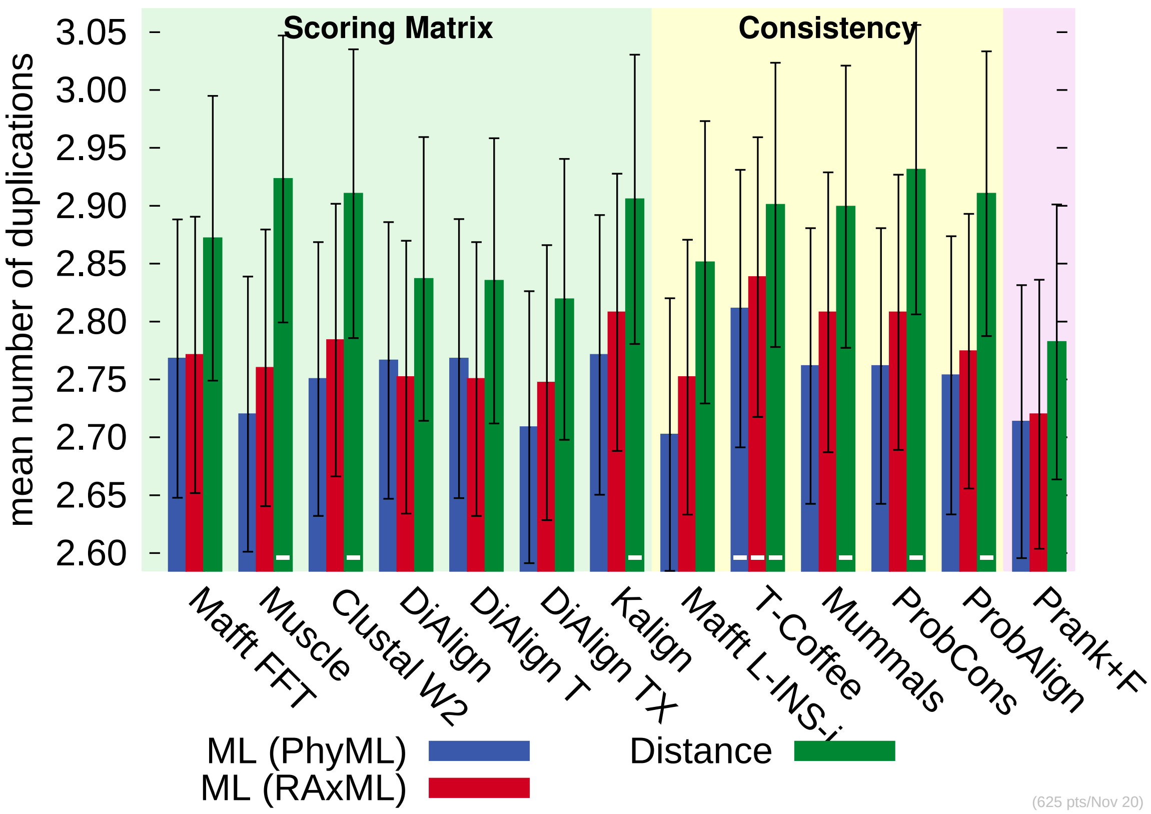

SFig. 9: Minimum duplication test, fungi dataset, amino-acid alignments and trees. The ranking of the alignment methods remains largely unchanged under two different ML implementations (RAxML, PhyML, both under JTT+Γ+I) and also under the LS tree building approach. Error bars correspond to ± 1 s.d. Significant difference from the best alignment program is denoted with a minus symbol at the basis of relevant bars (Wilcoxon double-sided test, P<0.01)

Partition analyses: sequence length

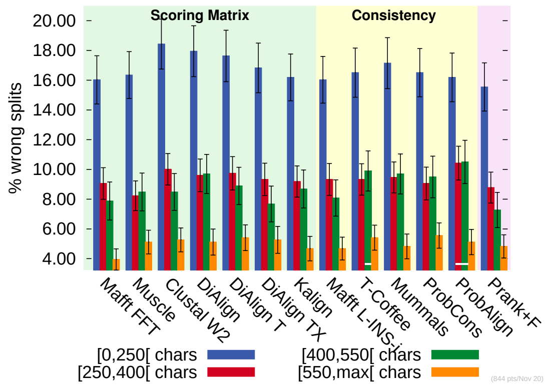

(a) Eukaryotes, Amino-acids

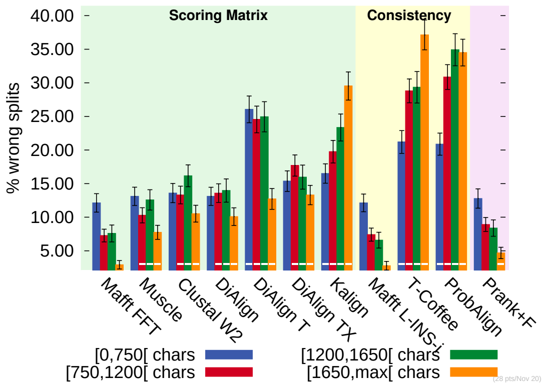

(b) Eukaryotes, Nucleotides

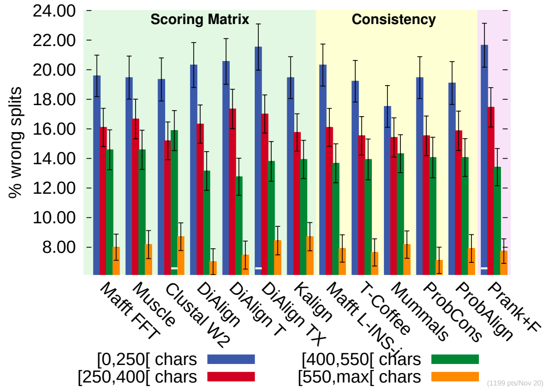

(c) Fungi, Amino-acids

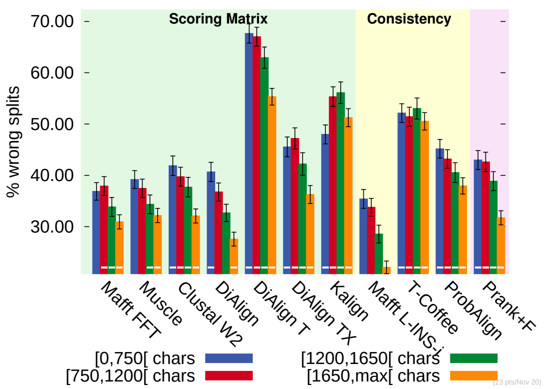

(d) Fungi, Nucleotides

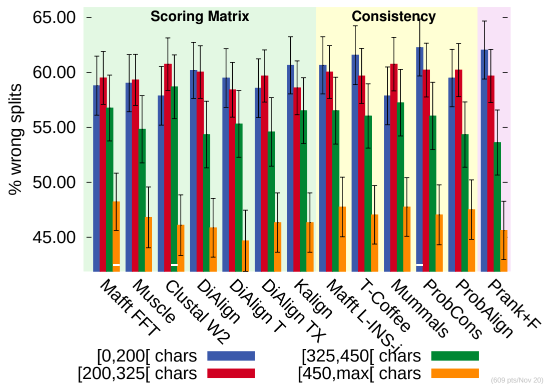

(e) Bacteria, Amino-acids

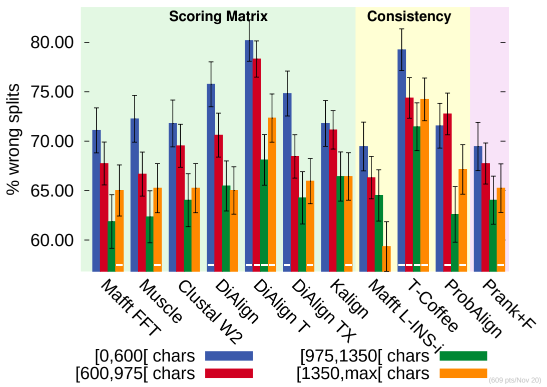

(f) Bacteria, Nucleotides

SFig. 10: Species-tree discordance test. The data was partitioned into 4 categories according to average sequence length. We observe that the relative performance of the programs is largely consistent between partitions. Error bars correspond to $\pm$ 1 s.d. Significant difference from best alignment program is denoted with a minus symbol at the basis of relevant bars (Wilcoxon double-sided test, P<0.01)

Partition analyses: sequence divergence

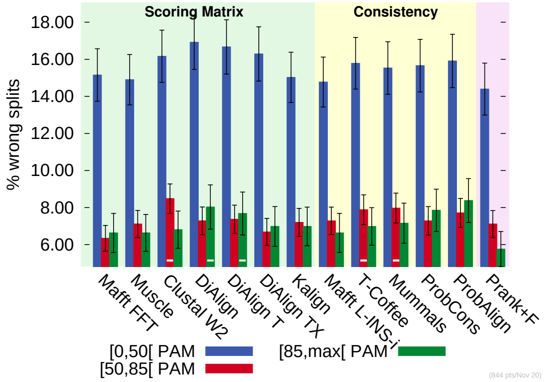

(a) Eukaryotes, Amino-acids

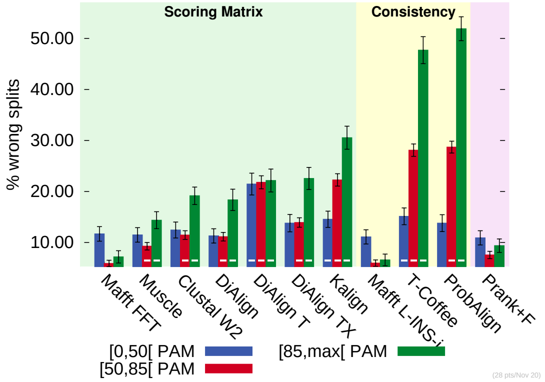

(b) Eukaryotes, Nucleotides

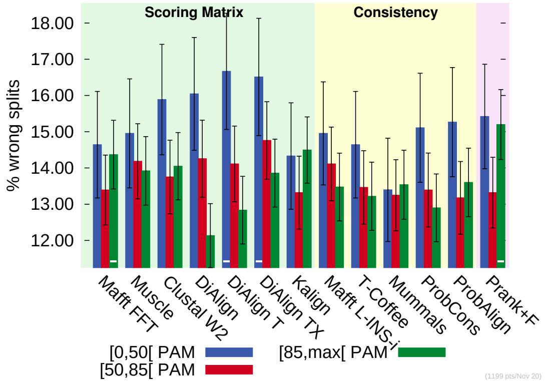

(c) Fungi, Amino-acids

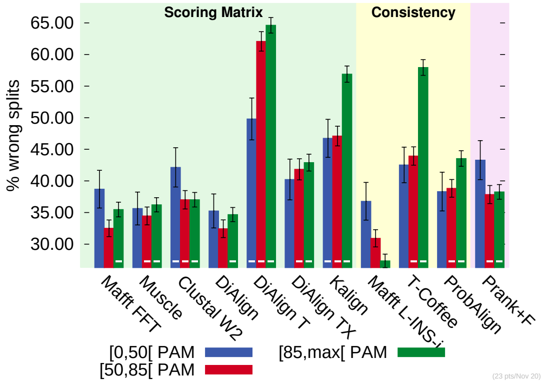

(d) Fungi, Nucleotides

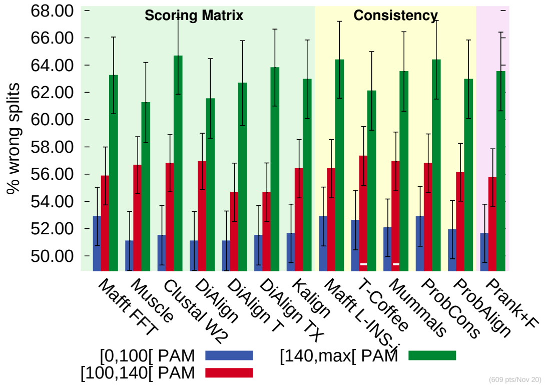

(e) Bacteria, Amino-acids

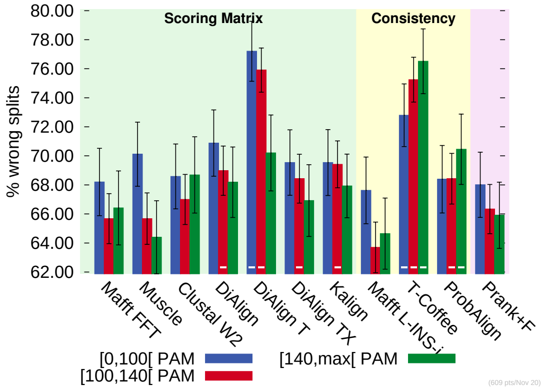

(f) Bacteria, Nucleotides

SFig. 11: Species-tree discordance test. The data was partitioned into 3 categories according to average divergence (in PAM units) in the pairs of sequence aligned by Mafft. We observe that the relative performance of the programs is largely consistent between partitions. Error bars correspond to $\pm$ 1 s.d. Significant difference from best alignment program is denoted with a minus symbol at the basis of relevant bars (Wilcoxon double-sided test, P<0.01).

Partition analyses: number of sequences

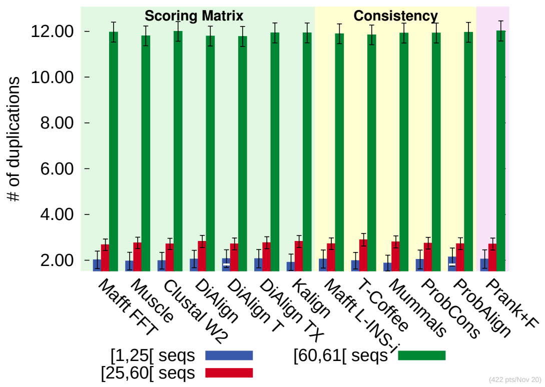

(a) Metazoa, Amino-acids

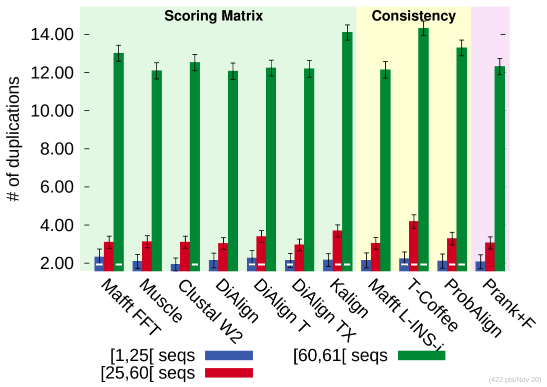

(b) Metazoa, Nucleotides

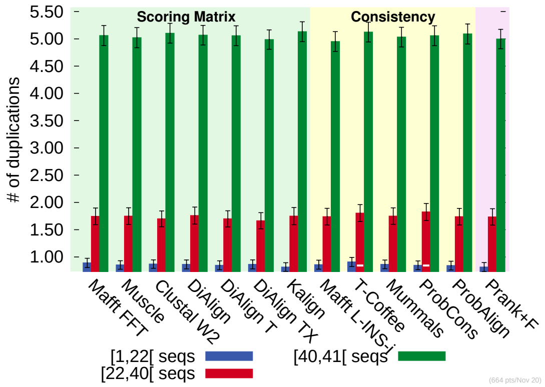

(c) Fungi, Amino-acids

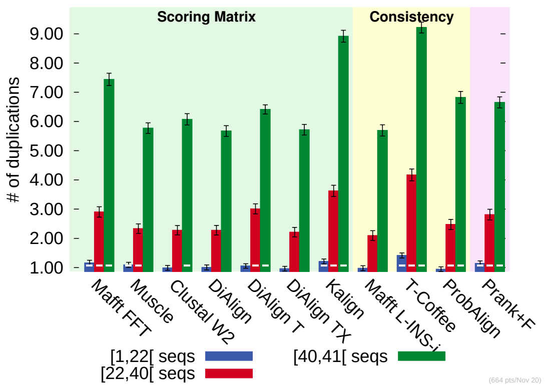

(d) Fungi, Nucleotides

SFig. 12: Minimum duplication test. The data was partitioned into 3 categories according to number of sequences in the input data. We observe that the relative performance of the programs is largely homogeneous. Error bars correspond to ± 1 s.d. Significant difference from best alignment program is denoted with a minus symbol at the basis of relevant bars(Wilcoxon double-sided test, P<0.01)

2.2 Guide Trees Make or Break Progressive Alignments

Sensitivity on guide tree specification (species-tree discordance test)

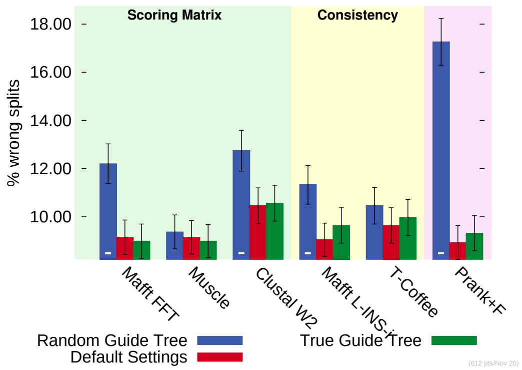

(a) Eukaryotes, Amino-acids

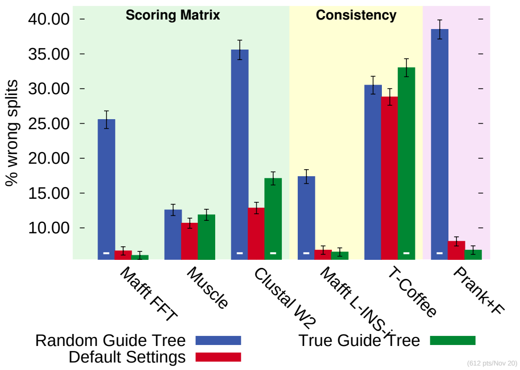

(b) Eukaryotes, Nucleotides

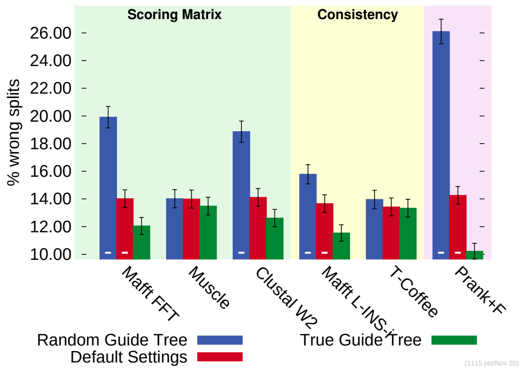

(c) Fungi, Amino-acids

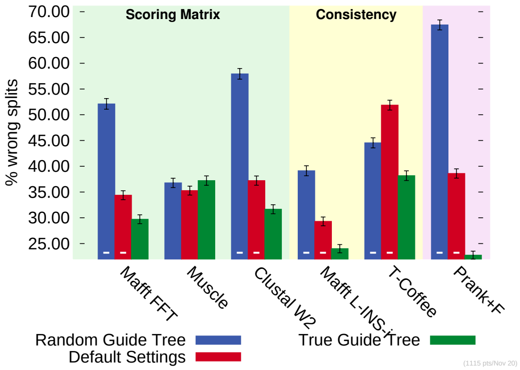

(d) Fungi, Nucleotides

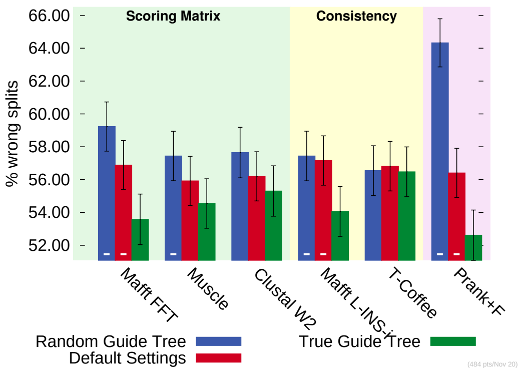

(e) Bacteria, Amino-acids

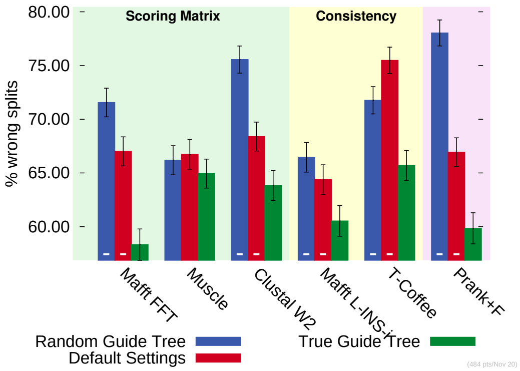

(f) Bacteria, Nucleotides

SFig. 13: Effect of guide tree specification in the species-tree discordance test. Error bars correspond to $\pm$ 1 s.d. Significant difference from best alignment program is denoted with a minus symbol at the basis of relevant bars (Wilcoxon double-sided test, P<0.01)

Comparison of guide tree accuracy (species-tree discordance test)

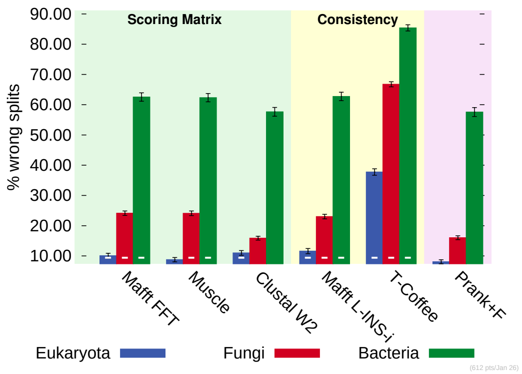

(a) Amino-acids

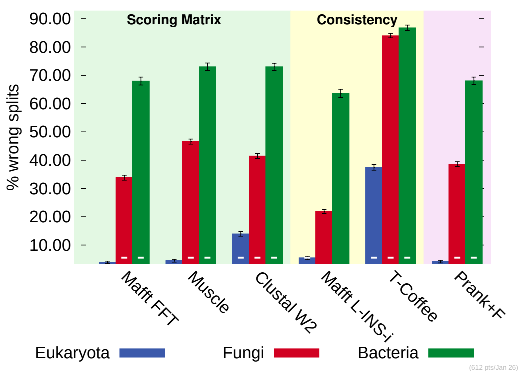

(b) Nucleotides

SFig. 14: Error bars correspond to $\pm$ 1 s.d. Significant difference from best guide tree construction method is denoted with a minus symbol at the basis of relevant bars (Wilcoxon double-sided test, P<0.01)

Combining the most accurate guide trees with other alignment programs (species-tree discordance test)

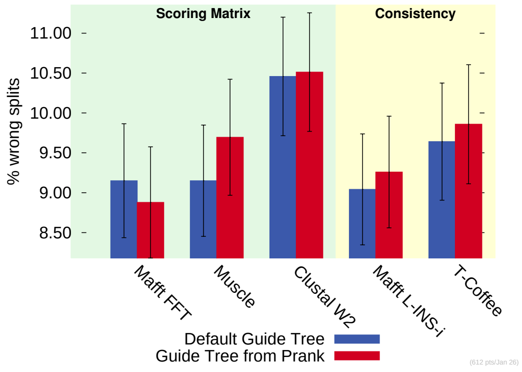

(a) Eukaryotes, Amino-acids

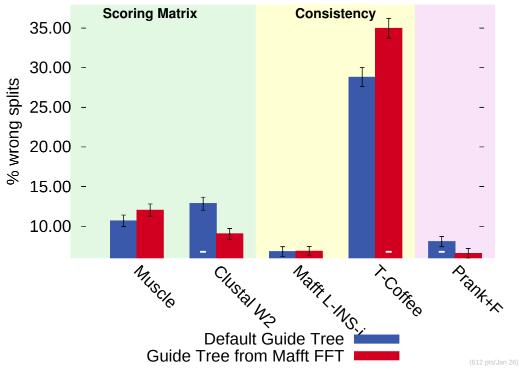

(b) Eukaryotes, Nucleotides

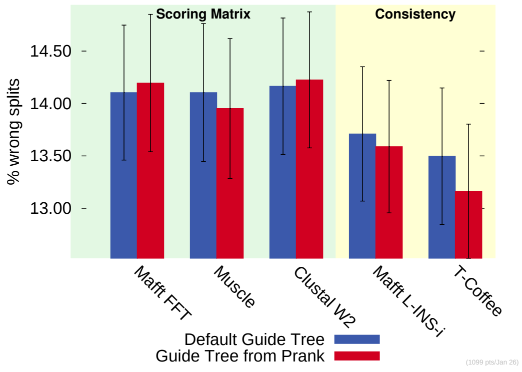

(c) Fungi, Amino-acids

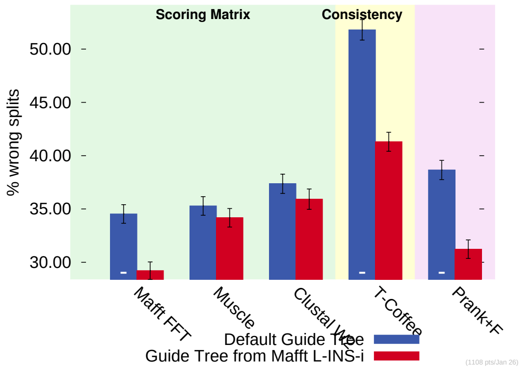

(d) Fungi, Nucleotides

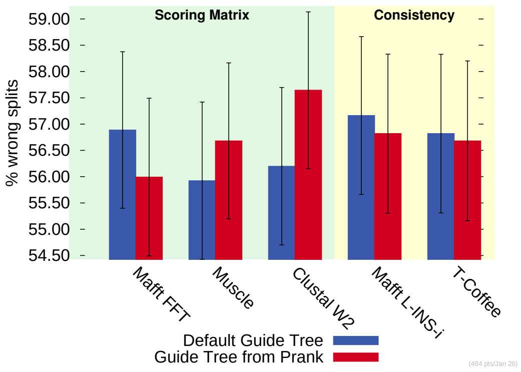

(e) Bacteria, Amino-acids

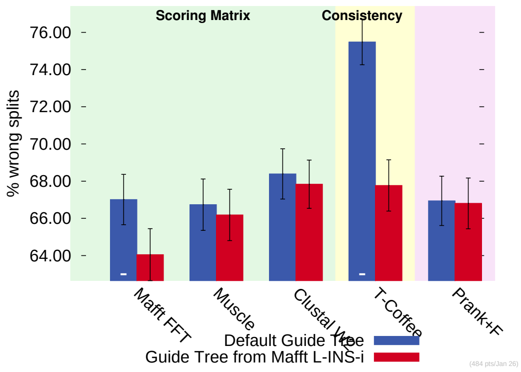

(f) Bacteria, Nucleotides

SFig. 15: SFig. 14 shows that the most accurate guide trees are reconstructed by Prank for amino-acids data, Mafft L-INS-i for bacterial and fungal nucleotide data, and Mafft FFT for eukaryotic nucleotide data. These plots show the effect of using the most accurate guide tree methods for other alignment programs. Error bars correspond to $\pm$ 1 s.d. Significant difference from best alignment program is denoted with a minus symbol at the basis of relevant bars (Wilcoxon double-sided test, P$<$0.01)

2.3 Are gap regions informative or should they be ignored?

- *Tests with Gap Parsimony Trees.* SFig. 16 shows the accuracy of gap placement among different programs, using parsimony on binary gap/non-gap characters (main methodology, see *Methods Summary: Tree Reconstruction*)

- *Tests with Gap Parsimony Trees (alternative methodology).* SFig. 17 shows the same test, but using an alternative methodology in extracting gap patterns in which repeated columns are *not* summarized (see *Methods: Alternative Tree Building Method*). This is to ensure that the results are not sensitive to the specifics of our tree building method.

- *Investigation of a Particular Gap Parsimony Tree Result: DiAlign-T on eukaryotic nucleotides.* In SFig. 16-17, Prank clearly outperformed the other packages with the exception of the DiAlign family in a few cases. Among those exceptions, the performance of DiAlign-T is especially striking on eukaryotic nucleotide data. To rule out a bug/error in our analysis, we investigated this case more closely. As it happens, in this particular dataset, DiAlign-T produced alignments that contained roughly four times more informative characters[1] (IC) than the alignments of Prank. And indeed, if we partition the input data according to the resulting number of ICs with Prank, we observe that the better performance of DiAlign-T is mainly concentrated in cases where the alignments of Prank lack IC (SFig. 18)

- *Effect of Exclusion of Gaps and Variable Regions.* SFig. 19-20 complete Fig. 4 in the main text with all other relevant experiments.

[1] In Wagner parsimony, a binary character is informative if each of the two states is shared by more than one taxon.

Tests with Gap Parsimony Trees

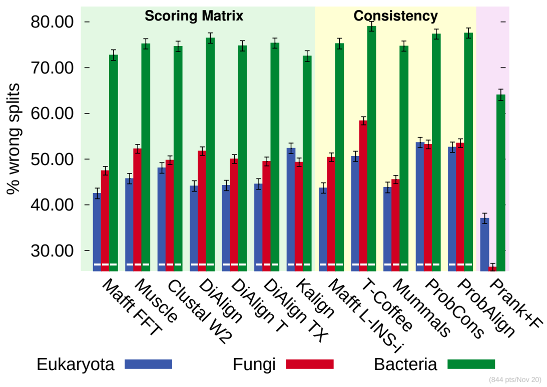

(a) Species-Tree Discordance, Amino-acids

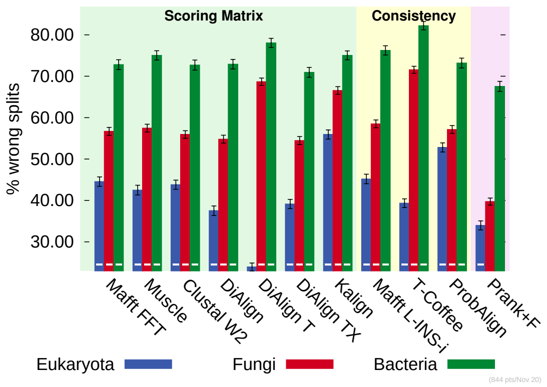

(b) Species-Tree Discordance, Nucleotides

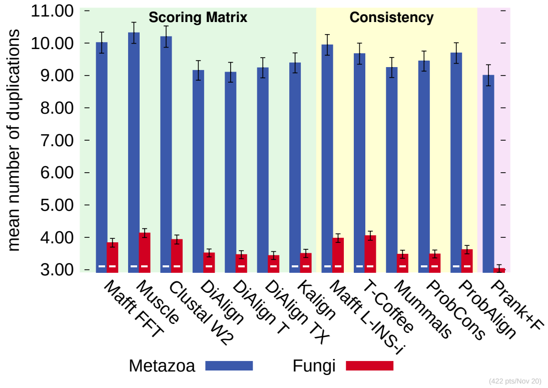

(c) Minimum Duplication, Amino-acids

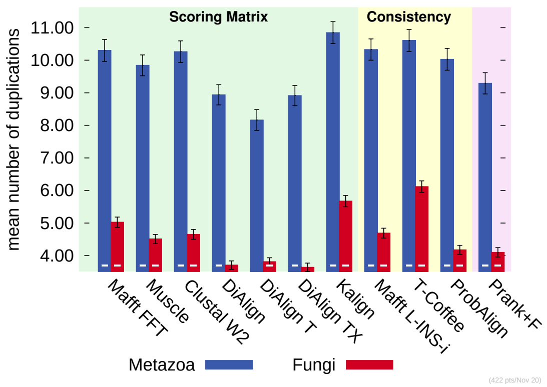

(d) Minimum Duplication, Nucleotides

SFig. 16: Accuracy of gap placement among different programs, using parsimony on binary gap/non-gap alphabet over entire alignment (main methodology, see *Methods*). Error bars correspond to ± 1 s.d. Significant difference from best program is denoted with a minus symbol at the basis of relevant bars (Wilcoxon double-sided test, P<0.01)

Gap Parsimony Trees (alternative methodology)

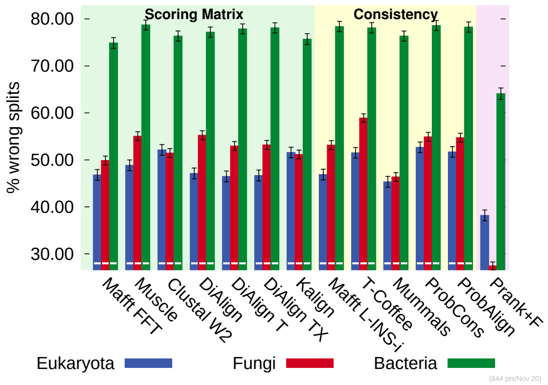

(a) Species-Tree Discordance, Amino-acids

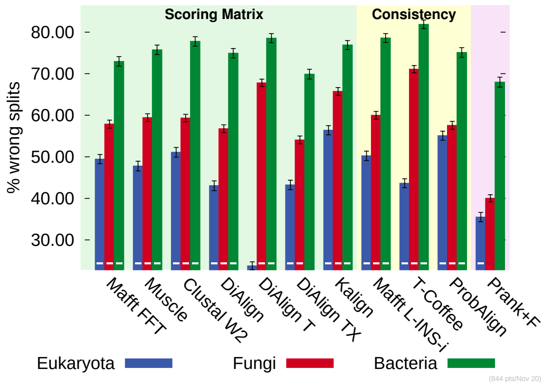

(b) Species-Tree Discordance, Nucleotides

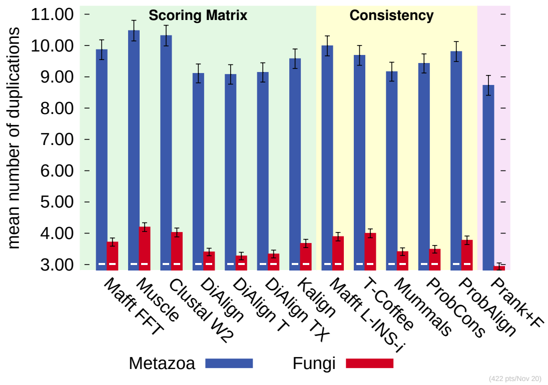

(c) Minimum Duplication, Amino-acids

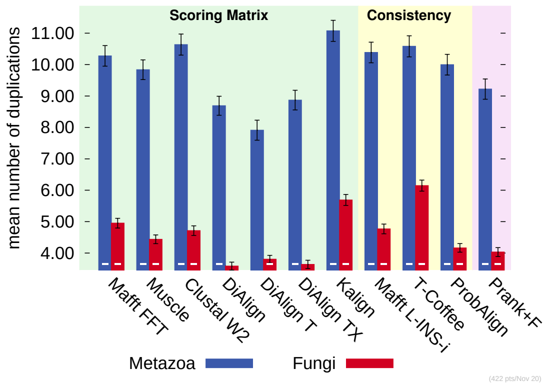

(d) Minimum Duplication, Nucleotides

SFig. 17: Accuracy of gap placement among different programs, using parsimony on binary gap/non-gap alphabet, but without combining repeated adjacent alignment columns (alternative methodology, see *Methods*). The results are highly consistent with SFig. 16, which suggests that they are robust to the specifics of the tree building method. Error bars correspond to $\pm$ 1 s.d. Significant difference from best program is denoted with a minus symbol at the basis of relevant bars (Wilcoxon double-sided test, P$<$0.01)

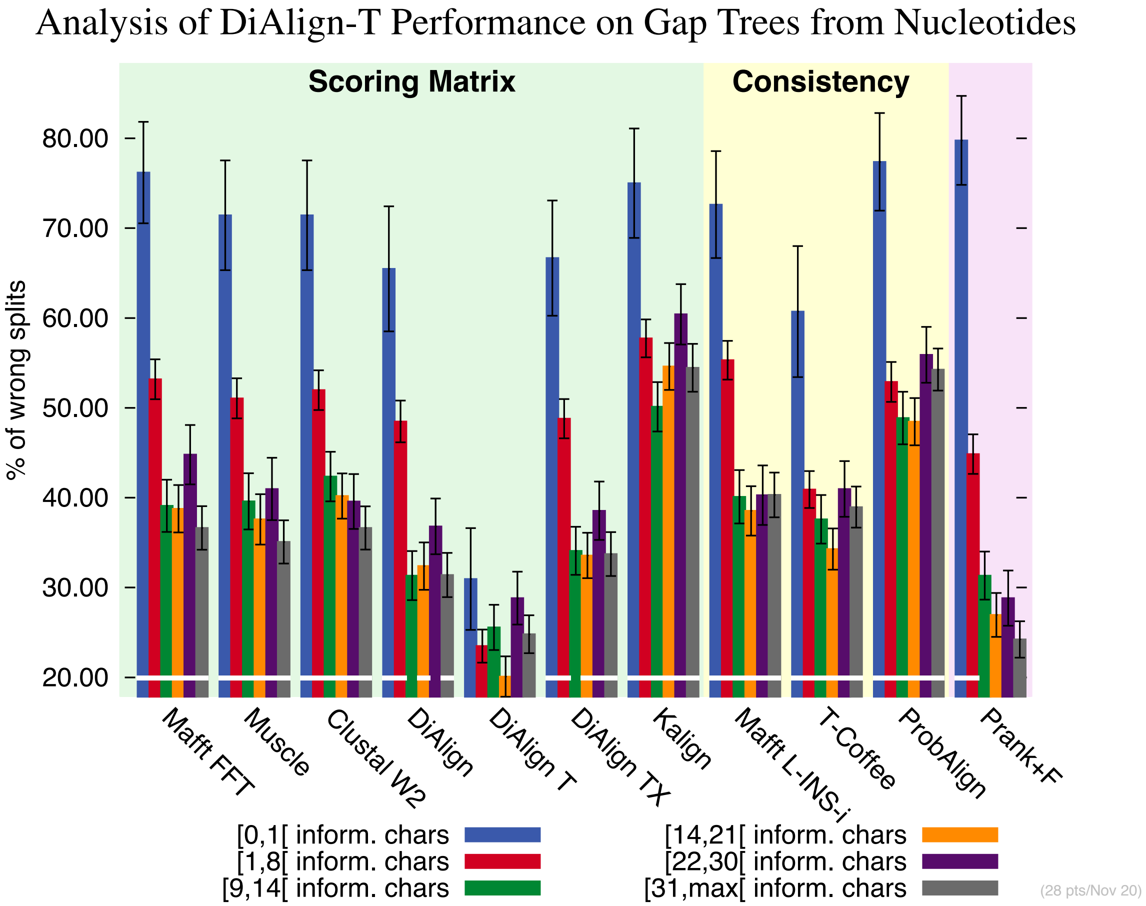

SFig. 18: Accuracy of gap parsimony trees, partitioned according to number of informative characters in Prank (Species-tree discordance test, eukaryotes, nucleotides). DiAlign T outperforms Prank mainly when the alignment of the latter have few informative characters. Error bars correspond to ± 1 s.d. Significant difference from best program is denoted with a minus symbol at the basis of relevant bars (Wilcoxon double-sided test, P<0.01)

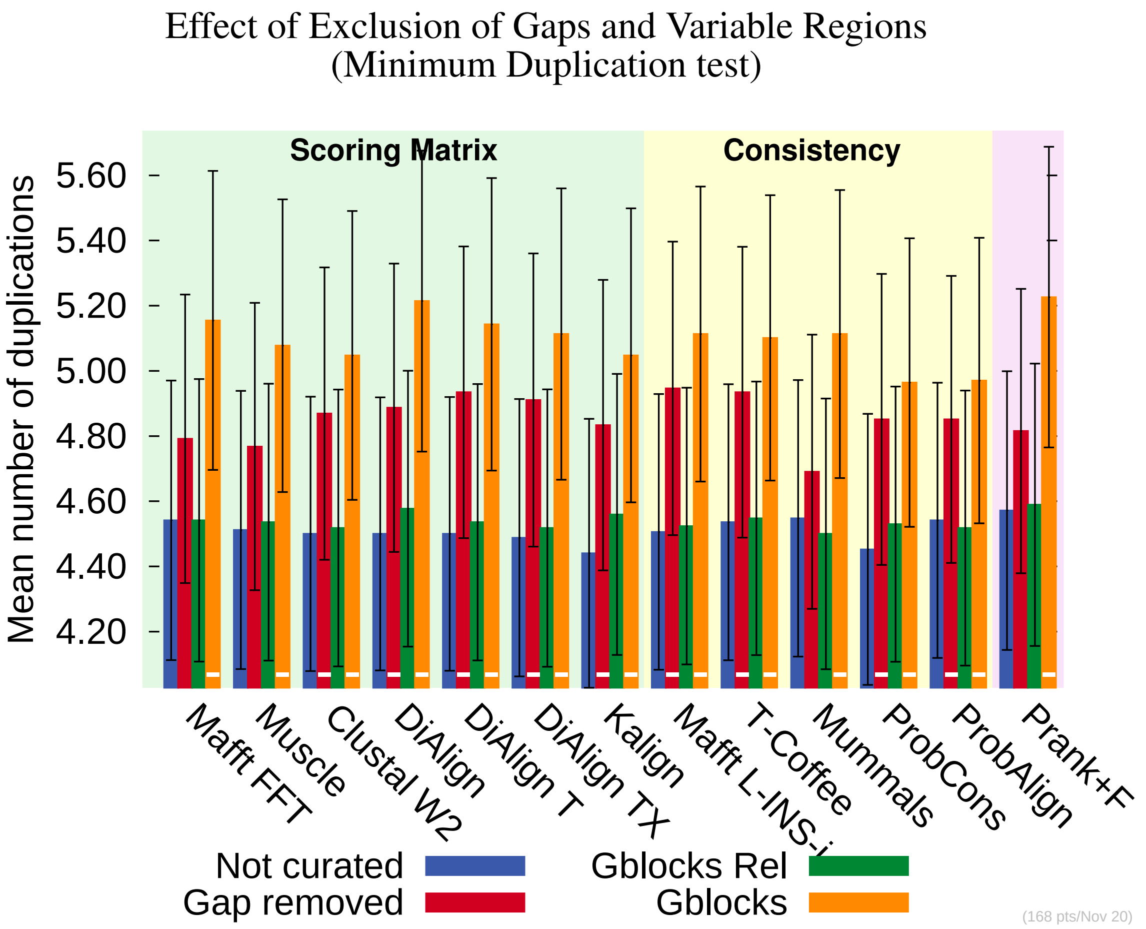

SFig. 19: Effect of gap exclusion and variable regions on minimum duplication test, back-translated amino-acid alignments, eukaryotes. Error bars correspond to ± 1 s.d. Significant difference from best filtering approach is denoted with a minus symbol at the basis of relevant bars (Wilcoxon double-sided test, P<0.01)

Effect of Exclusion of Gaps and Variable Regions
(Species-tree discordance test)

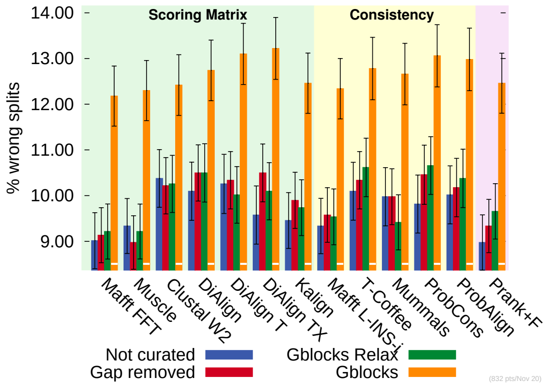

(a) Eukaryotes, Amino-acids

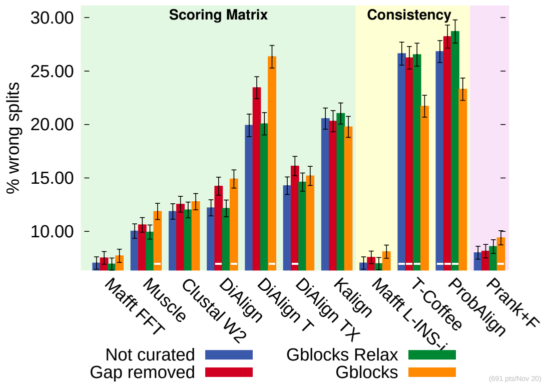

(b) Eukaryotes, Nucleotides

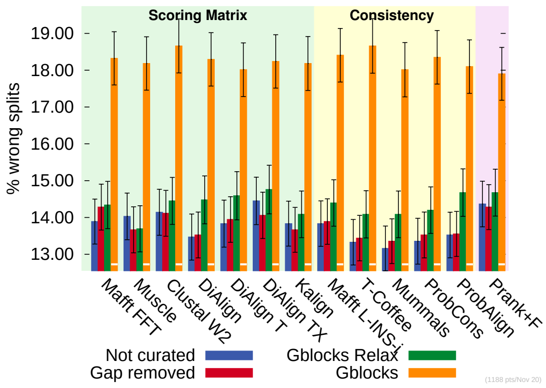

(c) Fungi, Amino-acids

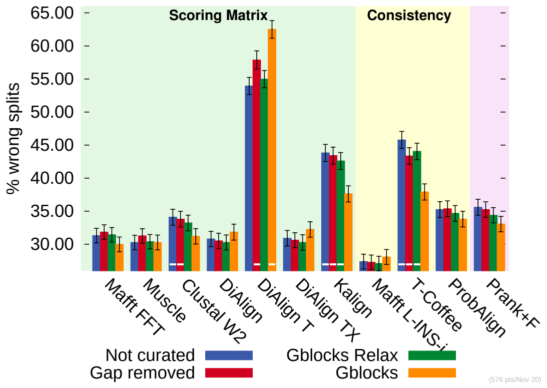

(d) Fungi, Nucleotides

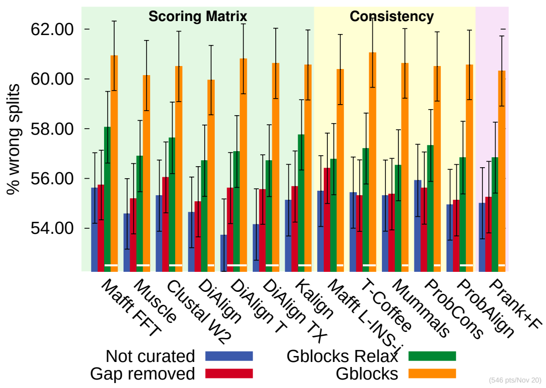

(e) Bacteria, Amino-acids

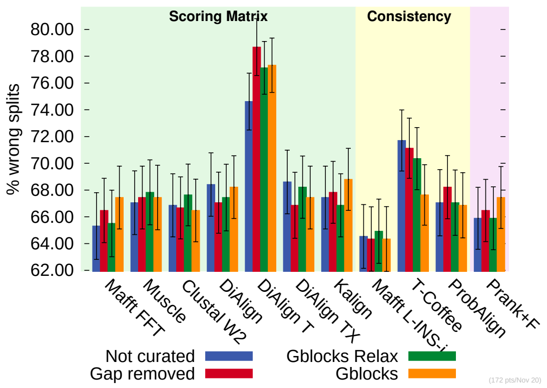

(f) Bacteria, Nucleotides

SFig. 20: Effect on species-tree discordance test of gap exclusion and variable regions. Error bars correspond to ± 1 s.d. Significant difference from best filtering approach is denoted with a minus symbol at the basis of relevant bars (Wilcoxon double-sided test, P<0.01)

2.4 What is the impact of alignment uncertainty on tree inference?

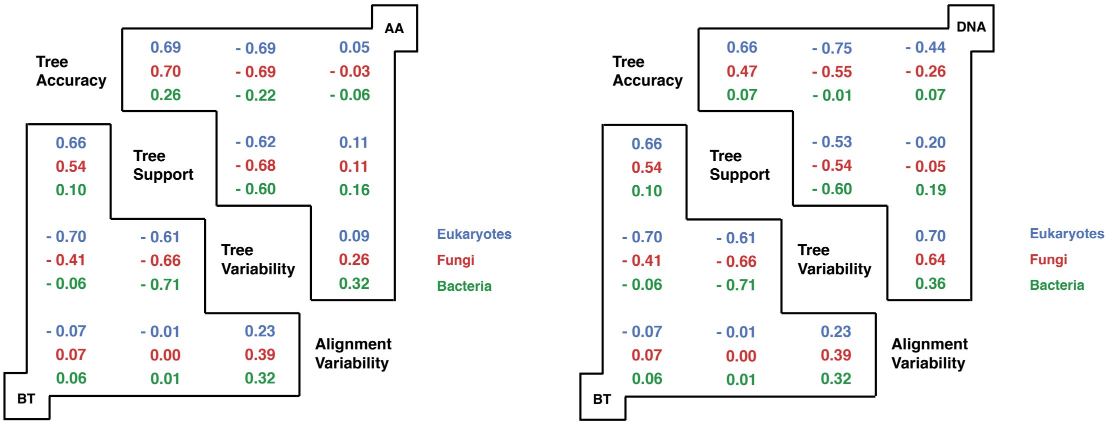

SFig. 21: Spearman's rank correlation estimates among various tree and alignment measures, from amino-acid (AA), nucleotide (DNA), and back-translated (BT) alignments. Alignment variability has a low correlation with tree accuracy, if any. In all cases, tree support (BS) is a better predictor for tree accuracy (TA) than alignment variability (AV) ($r_{BS,TA} > -r_{AV,TA}, P < 2 \cdot 10^{-2}$).

SFig 23 depicts the correlation matrices for the various tree and alignment measures discussed in the main tex. For the sake of completeness we give scatter plots (amino-acid: SFig 22, back-translation: SFig 23, nucleotides: SFig 24) corresponding to the correlation tables in SFig. 21.

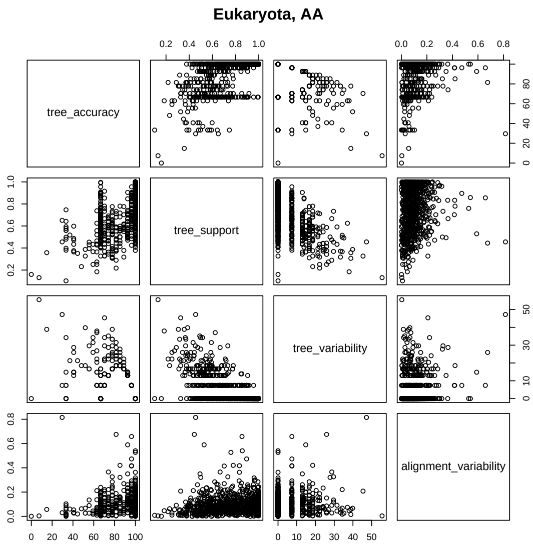

(a)

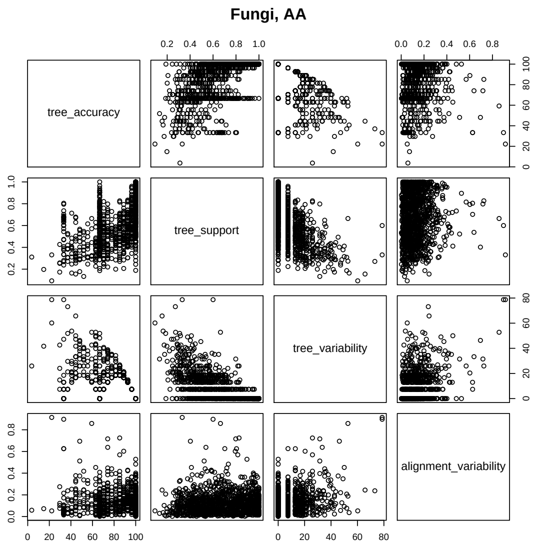

(b)

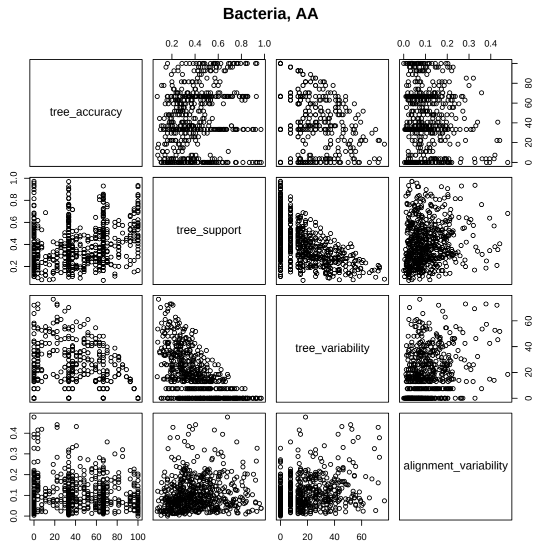

(c)

SFig. 22: Correlation plots for amino-acid data corresponding to the correlation table in SFig. 21

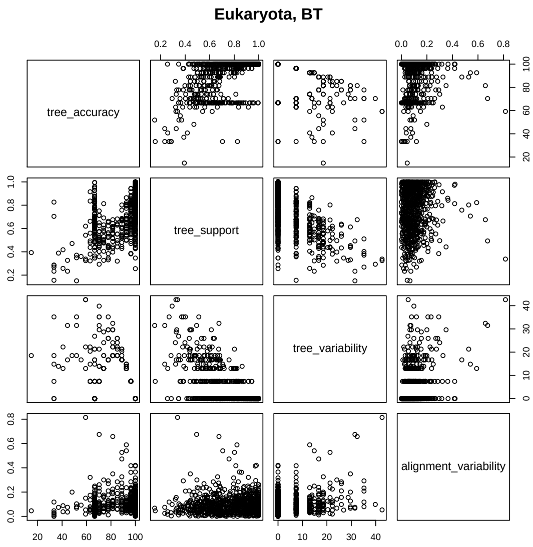

(a)

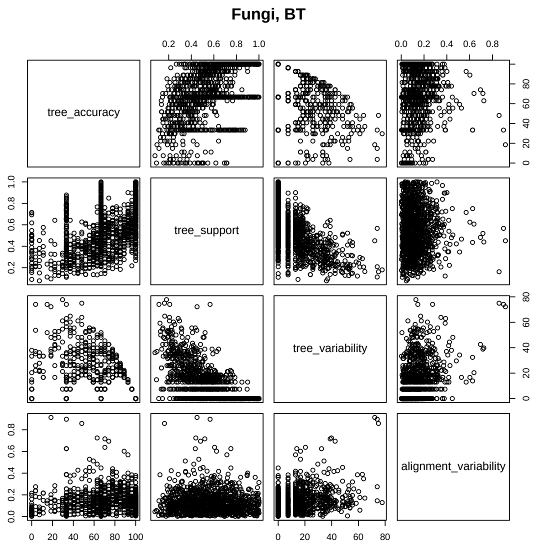

(b)

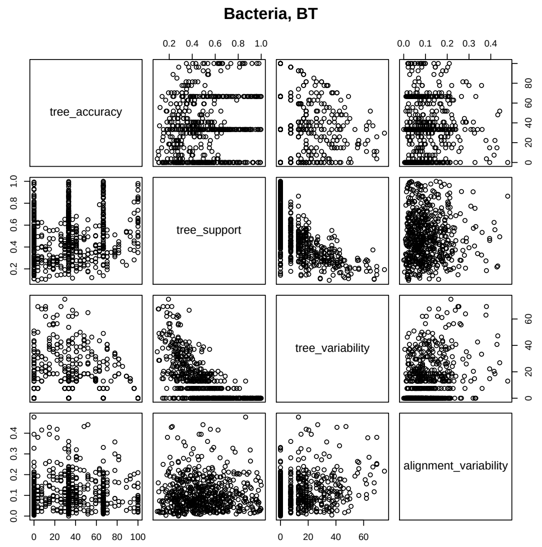

(c)

SFig. 23: Correlation plots for back-translated data corresponding to the correlation tables in SFig. 21.

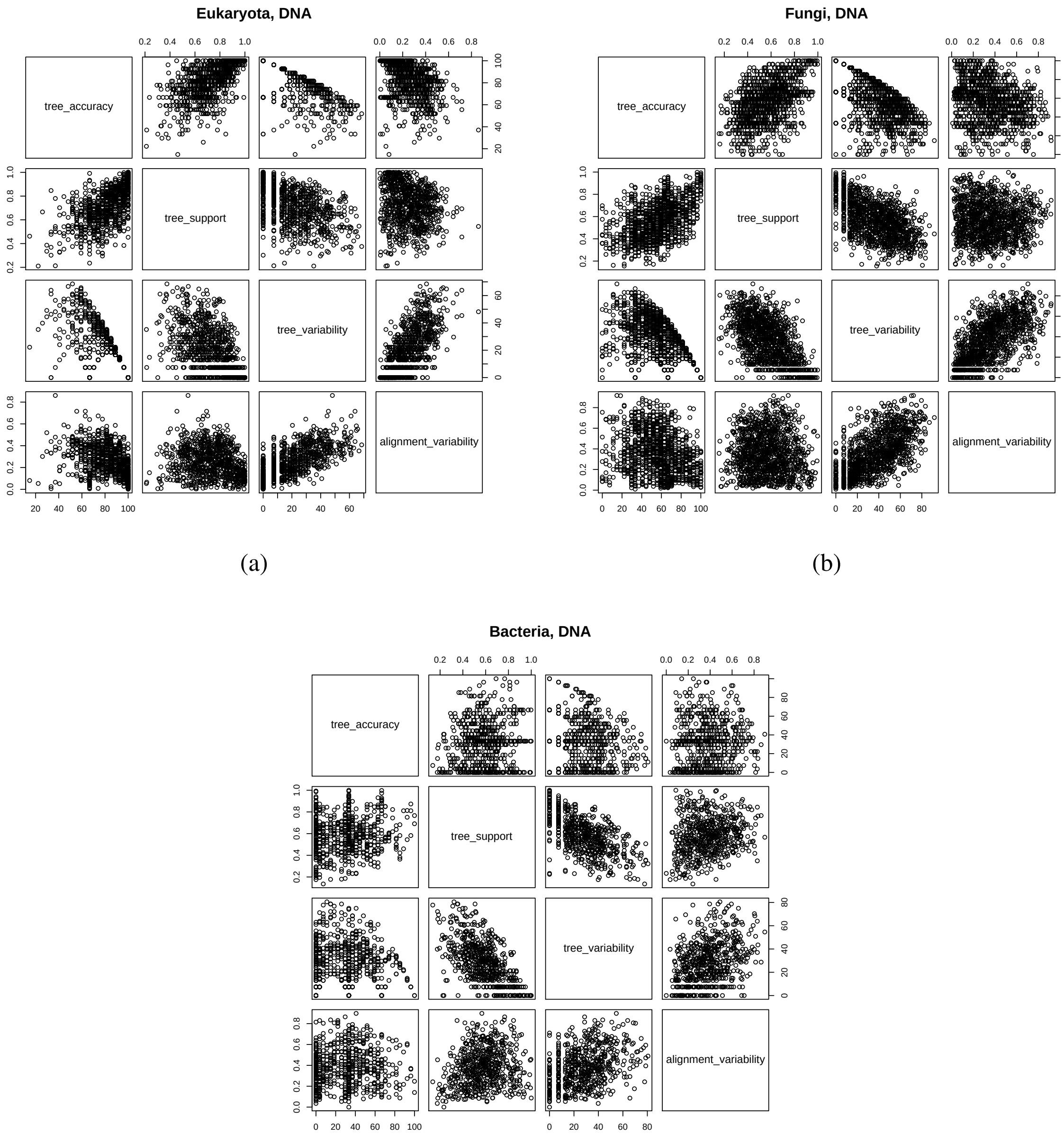

(a) (b) (c)

SFig. 24: Correlation plots for nucleotide data corresponding to the correlation table in SFig. 21.

3 Raw Data

All the files necessary to reproduce our study can be downloaded at

http://www.cbrg.ethz.ch/research/msa

Aligned and unaligned sequence data are given in Fasta format. Phylogenetic trees are stored in Newick format. The data is organized using the directory-structure

/testtype/taxonomic_group/problem_nb/data_type/experiment/msa_package/

The placeholders have the following meaning:

- *testtype* The results of the minimum duplication test (MD) and of the species-tree discordance test (SD) are under the two separate directories "SD" and "MD".
- *taxonomic_groups* The two test were each run on different taxonomic groups. For MD the groups are "Fungi" and "Metazoa". For SD they are "Bacteria", "Eukaryota" an "Fungi".
- *problem_nb* The problem number refers to a particular set of sequences sampled from a taxonomic group. The corresponding reference topologies for SD (used to score reconstructed trees) are stored at this directory-level.
- *data_type* This level separates the results from the amino-acid "AA", nucleotide "DNA" and back-translated "BT" alignments. The unaligned AA and nucleotide sequences are stored under the corresponding directories.
- *experiment* This level separates the results from the original alignments "OA", from the alignments curated by Gblocks (stringent settings "G", relaxed settings "GR"), from the alignments with removed gap-columns "NG" and from the bootstrap alignments "BS". Note that BS has only been performed for SD.

- *msa_package* This directories contain the results for each alignment package, i.e. a multiple sequence alignment and the trees reconstructed from the alignment. Note that the two gap parsimony tree variants are named "ParsimonyND" for when repeated neighbouring characters are removed and "Parsimony" otherwise (see *Methods*)
